# Tobacco harm reduction: an alternative cessation strategy for inveterate smokers

**DOI:** 10.1186/1477-7517-3-37

**Published:** 2006-12-21

**Authors:** Brad Rodu, William T Godshall

**Affiliations:** 1 Professor of Medicine and Endowed Chair, Tobacco Harm Reduction Research, School of Medicine, University of Louisville, KY, USA; 2 Founder and Executive Director, Smokefree Pennsylvania, Pittsburgh, PA, USA

## Abstract

According to the Centers for Disease Control and Prevention, about 45 million Americans continue to smoke, even after one of the most intense public health campaigns in history, now over 40 years old. Each year some 438,000 smokers die from smoking-related diseases, including lung and other cancers, cardiovascular disorders and pulmonary diseases.

Many smokers are unable – or at least unwilling – to achieve cessation through complete nicotine and tobacco abstinence; they continue smoking despite the very real and obvious adverse health consequences. Conventional smoking cessation policies and programs generally present smokers with two unpleasant alternatives: quit, or die.

A third approach to smoking cessation, tobacco harm reduction, involves the use of alternative sources of nicotine, including modern smokeless tobacco products. A substantial body of research, much of it produced over the past decade, establishes the scientific and medical foundation for tobacco harm reduction using smokeless tobacco products.

This report provides a description of traditional and modern smokeless tobacco products, and of the prevalence of their use in the United States and Sweden. It reviews the epidemiologic evidence for low health risks associated with smokeless use, both in absolute terms and in comparison to the much higher risks of smoking. The report also describes evidence that smokeless tobacco has served as an effective substitute for cigarettes among Swedish men, who consequently have among the lowest smoking-related mortality rates in the developed world. The report documents the fact that extensive misinformation about ST products is widely available from ostensibly reputable sources, including governmental health agencies and major health organizations.

The American Council on Science and Health believes that strong support of tobacco harm reduction is fully consistent with its mission to promote sound science in regulation and in public policy, and to assist consumers in distinguishing real health threats from spurious health claims. As this report documents, there is a strong scientific and medical foundation for tobacco harm reduction, and it shows great potential as a public health strategy to help millions of smokers.

## I. Background

According to the Centers for Disease Control and Prevention (CDC), about 45 million Americans continue to smoke [1], even after one of the most intense public health campaigns in history, now over 40 years old. Some 438,000 smokers die from smoking-related diseases each year, including lung and other cancers, cardiovascular disorders and pulmonary diseases [2].

There is clear evidence that smokers of any age can reap substantial health benefits by quitting. In fact, no other single public health effort is likely to achieve a benefit comparable to large-scale smoking cessation. Surveys document that most smokers would like to quit, and many have made repeated efforts to do so. However, conventional smoking cessation approaches require nicotine-addicted smokers to abstain from tobacco and nicotine entirely (as discussed later, use of nicotine replacement medications is limited to 10–12 weeks, per labels required by federal regulations). Many smokers are unable – or at least unwilling – to achieve this goal, and so they continue smoking in the face of impending adverse health consequences. In effect, the status quo in smoking cessation presents smokers with just two unpleasant alternatives: quit or die.

There is a third choice for smokers: tobacco harm reduction. It involves the use of alternative sources of nicotine, including modern smokeless tobacco (ST) products, by those smokers who are unable or unwilling to quit tobacco and nicotine entirely. The history of tobacco harm reduction may be traced back to 1974, with the publication of a special article in the *Lancet *by British tobacco addiction research expert Michael A.H. Russell [3]. Citing the "high dependence-producing potency and the universal appeal of the effects of nicotine" on smokers, Russell likened "harsher restrictive measures" and "intensification" of anti-smoking efforts to "flogging a dead horse harder." Russell believed that "the goal of abstinence and the abolition of all smoking is unrealistic and doomed to fail."

Six years later Russell's research group compared nicotine absorption rates from various tobacco products, which led them to suggest that nasal snuff use could serve as an effective substitute for cigarette smoking [4]. This article was cited shortly thereafter by a short letter in a leading American medical journal [5]. Russell et al published follow-up studies on nasal snuff in 1981 [6] and on an oral ST product in 1985 [7]. Lynn Kozlowski, a prominent American smoking and nicotine addiction expert at Penn State University, noted in 1984 and 1989 that ST products conferred fewer risks to users and therefore might serve as effective substitutes for cigarettes [8,9]. In 1994 oral pathologist Brad Rodu and epidemiologist Philip Cole from the University of Alabama at Birmingham made quantitative comparisons of the risks from oral ST use and smoking in a series of studies [10-13]. Some of that work was summarized in a 1995 ACSH publication [14].

A substantial body of research over the past decade has been transformed into the scientific and medical foundation for tobacco harm reduction, the substitution of safer sources of nicotine, including tobacco products, by those smokers who are unable or unwilling to achieve nicotine and tobacco abstinence. In 2001 the Institute of Medicine, a subsidiary of the National Academy of Sciences, provided a now widely accepted definition of a harm reduction product as "harm reducing if it lowers total tobacco related mortality and morbidity even though use of that product may involve continued exposure to tobacco related toxicants" [15]. The purpose of this report is to review the evidence for tobacco harm reduction.

## II. The status quo: cigarette smoking

### A. Prevalence

At first glance, the United States (U.S.) appears to be the quintessential example of the slow but substantial decline of cigarette smoking in the developed world. Smoking prevalence in the U.S. has decreased since at least the mid-1960s, following landmark reports from the Royal College of Physicians of London in 1962 and the U.S. Surgeon General in 1964. Smoking among men was 52% in 1965 [16], dropping to 23% by 2004 [1]. Prevalence among women declined from 34% in 1965 to 19% in 2004. In 1965, only 44% of American adults had never smoked and 14% were former smokers; by 2004, those percentages had increased to 58% and 21% respectively.

But declining prevalence overshadows the fact that, with population growth, the absolute number of smokers in the U.S. remained relatively constant at 45 to 50 million over the entire period. Heavily-addicted, or inveterate, smokers are resistant to conventional cessation strategies emphasizing tobacco and nicotine abstinence. Today's smoking population has a higher proportion of heavy smokers than in the past, and the National Cancer Institute (NCI)-funded Community Intervention Trial for Smoking Cessation underscores the challenges facing them [17]. Perhaps the most intensive cessation trial ever conducted, this 4-year effort had no effect on cessation among heavy smokers. The published report called the intervention "disappointing but consistent with the findings of most other community studies...", and it described heavy smokers as "more resistant to change. Reaching these smokers may require new clinical programs and public policy changes."

### B. Health effects

Cigarette smoking remains the single most important avoidable cause of death in the developed world. The CDC reports that smoking is responsible for 438,000 deaths in the U.S. annually [2], a figure which has changed little over the last 15 years.

Cigarette smoking was responsible for a large proportion of the increase in cancer mortality in the second half of the 20^th ^Century, a trend with important social consequences, including the widespread misperception that the U.S. was being consumed by a "cancer epidemic" caused by environmental pollution and industrial chemicals. In fact, the "epidemic" consisted almost exclusively of one disease, lung cancer, and was due to one lifestyle factor, cigarette smoking. A retrospective analysis of mortality statistics revealed that, if lung cancer is excluded, the mortality rate from all other forms of cancer combined has declined continuously since 1950 [18].

The first reports linking lung cancer to cigarette smoking were published over 50 years ago [19,20]. In 2006 there will be 175,000 new cases of lung cancer in the U.S., with a five-year survival rate of just 15% [21]. The CDC estimates that smoking causes 142,000 deaths per year from lung cancer [2]. Smoking is a risk factor for other malignancies, including cancers of the oral cavity and pharynx, larynx, esophagus, stomach, bladder, kidney, pancreas, uterine cervix and leukemia [2].

According to the CDC, smoking causes 132,000 deaths per year from cardiovascular diseases, including heart attacks, strokes, atherosclerosis and aortic aneurysms [2]. Smoking is also causes 103,000 deaths per year from pulmonary diseases such as pneumonia, influenza, bronchitis and chronic airway obstruction [2].

While many Americans are aware that cigarette smoking causes cancer, cardiovascular and respiratory diseases, most are not aware that it also increases risks for neurological disorders, reproductive complications, cataracts and other eye diseases, premature aging of the skin, osteoporosis and other orthopedic and rheumatologic problems, psychiatric disorders and surgical complications [22]. Recent studies have also linked smoking to the development of type 2 diabetes [23-25].

### C. Stagnation

As Russell noted 30 years ago, "There is little doubt that if it were not for the nicotine...people would be little more inclined to smoke than they are to blow bubbles or light sparklers" [3] . Nicotine fulfills all the criteria of an addictive agent, including psychoactive effects, drug-reinforced behavior, compulsive use, relapse after abstinence, physical dependence, and tolerance. Nicotine stimulates specialized receptors in the brain which produce both euphoric and sedative effects. It has been known for many years that nicotine shares many features of drug dependence with opioids, alcohol and cocaine. This includes similar disappointing patterns of relapse [26].

It is for this reason that most attempts at smoking cessation are not successful, despite the fact that the majority of smokers are aware that smoking is harmful to their health, and so would like to quit. It is clear that most smokers would rather quit on their own, and 90% of successful quitters use self-help methods because of limited access to and cost of formal cessation programs [27].

Formal cessation programs have existed for decades and have grown more complex and sophisticated, but relapse rates remain very high. According to a 2006 National Institutes of Health (NIH) Consensus Conference on Tobacco Use, "70 percent [of smokers] want to quit and 40 percent make a serious quit attempt each year, but fewer than 5 percent succeed in any given year" [28]. The conference press release went on to make an astounding admission, "Effective tobacco cessation interventions are available and could double or triple quit rates..." This means that fewer than 15% of existing smokers, no more than 7 million, would be successful with maximum application of existing cessation strategies. The consensus statement failed to answer a vital question: What can be done for the remaining 40 million adult smokers? The rest of this report will review the scientific rationale and evidence for tobacco harm reduction as an alternative for these smokers.

## III. Smokeless tobacco use

### A. Introduction

The tobacco plant is native to the Western hemisphere, and the use of tobacco in smokeless forms (placed in the mouth or inhaled as a powder through the nose) predates the arrival and exploration of the West by Europeans. According to the historian Jan Rogozinski, the most common manufactured tobacco product in Europe until the early 1800s was a compressed plug or cake [29]. This product was relatively simple to produce and was amenable to transport and storage. The plug could be cut into large pieces for chewing, grated into smaller pieces for smoking, or ground into a fine powder for nasal inhalation. Smokeless forms were the favored method of use because a day's supply could be carried and conveniently used in industrial and agricultural work settings.

ST was the dominant form of tobacco used in the U.S. until early in the 20^th ^century [29]. Developments in tobacco cultivation, curing and manufacturing, along with the invention of the safety match, resulted in the increased popularity of cigarettes. In addition, at the beginning of the 20^th ^century tobacco spit inaccurately was believed to transmit tuberculosis, so bans on public spitting and spittoons resulted in a decline in ST use. The transmission of tuberculosis now has been understood for decades, and it does not include expectoration [30].

Use of all types of ST traditionally has been most prevalent in Southern states and in rural areas throughout the U.S.

### B. Types of ST

As described below, ST is currently used by only a small proportion of American tobacco users. This is one reason that most Americans, including smokers, know almost nothing about ST products, or – even worse – are completely misinformed about even basic product characteristics. Thus, it is important to understand what these products are and how they are used.

ST products are not burned but instead are placed in the cheek or between the lip and gum. ST is used in many countries around the world, including those in the Middle East and on the Indian subcontinent. However, ST products in those regions are considerably different from those used in the West. For example, in India ST products are made by individual farmers and small companies with little control over fermentation and curing, which affects the production of potential carcinogens called tobacco-specific nitrosamines (TSNAs) [31]. In India ST is often combined with betel leaf (*Piper betle*), sliced areca nut (*Areca catechu*) and/or powdered agricultural lime [32], additives that enhance the toxicity as well as the psychotropic effect of tobacco [33,34]. In addition, Indian ST users often smoke concurrently, which complicates efforts to assess the health effects of ST use [35,36].

This report will focus on ST products used in Western societies, mainly the U.S. and Sweden. But ST is not a homogeneous category, even in these countries. Three traditional types of ST are used in the U.S.: powdered dry snuff, loose leaf chewing tobacco and moist snuff, and it is important to understand the differences among them with respect to their manufacturing and characteristics, the populations that consume them, and the consequential health risks, especially mouth cancer.

#### Powdered dry snuff (Figure 1)

**Figure 1:**
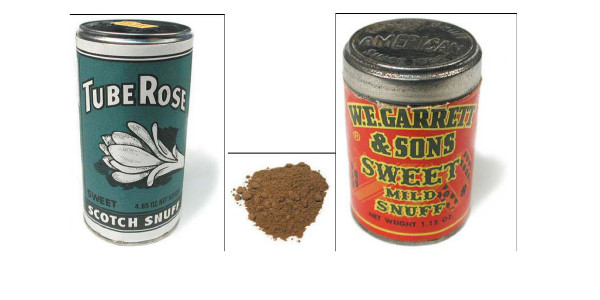
Powdered dry snuff.

Dry snuff is made from fermented, fire-cured tobacco that is pulverized into powder. Nasal inhalation of dry snuff was widely practiced in Europe in the 17^th ^and 18^th ^centuries but declined thereafter [37]. Manufacturers in Germany and the U.K. still provide an array of flavored dry snuff products for a small number of contemporary users in those countries. In the U.S. powdered dry snuff, also called dental or Scotch snuff, is sold in small canisters. Since the early 1800s it has been used primarily by women in Southern states [29,38], who place the powder on the gum or between the gum and cheek. However, use of dry snuff is declining, and sales have fallen 67% in the past 15 years [39].

#### Loose leaf chewing tobacco (Figure 2)

**Figure 2:**
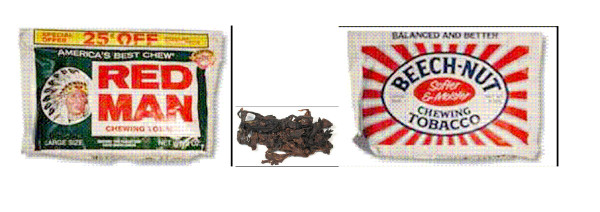
Loose leaf chewing tobacco.

Loose-leaf chewing tobacco consists of air-cured leaf tobacco from Pennsylvania and Wisconsin that is shredded, coated with sweet flavoring solutions and packaged in foil-lined pouches. It is consumed primarily by men in the U.S., commonly in conjunction with outdoor activities. Chewing tobacco is typically used in large volumes, resulting in the archetypical golf ball-sized bulge in the user's cheek and large quantities of saliva that users usually expectorate. Consequently, the popularity of this product has waned, with consumption declining gradually over the past century, dropping by about 44% in just the last 15 years [39].

#### Moist snuff (Figure 3)

**Figure 3:**
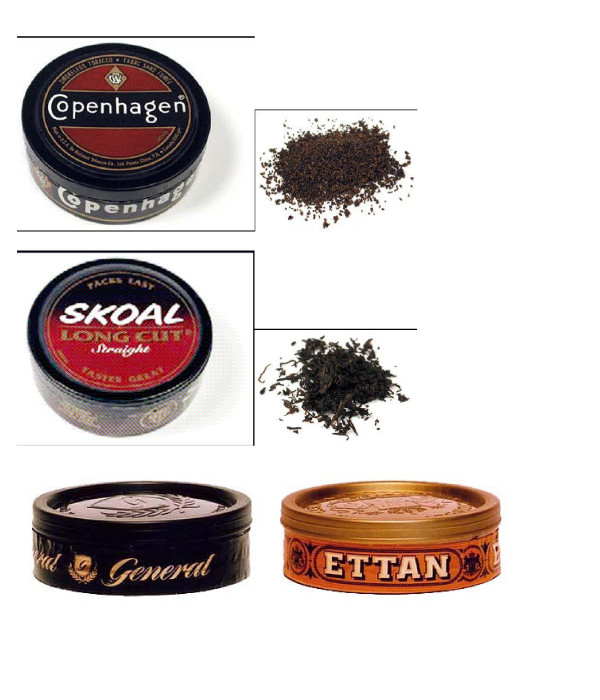
Moist snuff.

Moist snuff consists of fire- and air-cured dark tobaccos that are finely cut or ground. It is packaged in round containers, and the user compresses a "pinch" between the thumb and forefinger and places it inside the lip. Much less bulky than loose leaf chewing tobacco, moist snuff produces less saliva, but expectoration is still common. It is now the most popular form of ST in the U.S.; sales of this product increased by 66% over the past 15 years [39].

In addition to the U.S., there is a long tradition of moist snuff use in Scandinavia, especially in Sweden, where "snus" (the generic term for moist snuff in Swedish, pronounced "snoose") is essentially the only type of ST product in use [40]. There are differences in how American and Swedish moist snuff products are manufactured. Traditional American products undergo fermentation, which imparts characteristic flavors but in the past resulted in higher concentrations of unwanted bacterially mediated by-products, especially TSNAs and nitrite. In Sweden, moist snuff is subjected during manufacturing to a heat treatment akin to pasteurization, yielding virtually sterile products containing very low levels of TSNAs. However, manufacturing refinements over the past 25 years have resulted in lower TSNAs in both Swedish and American products. A 1997 report by the Swedish National Board of Health and Welfare reported that TSNA concentrations in both Swedish and American ST brands had declined substantially [41]. The report concluded: "Recent data suggest that the differences [in TSNA levels reported in American and Swedish ST] have grown smaller, and that it is now questionable to make a sharp distinction between use of American and Swedish moist snuff when assessing risks – at least where TSNA content is concerned."

A separate section of this report will discuss how the high prevalence of snus use in Sweden has played an important role in the low prevalence of smoking, especially among men.

#### Modern ST products (Figure 4)

**Figure 4:**
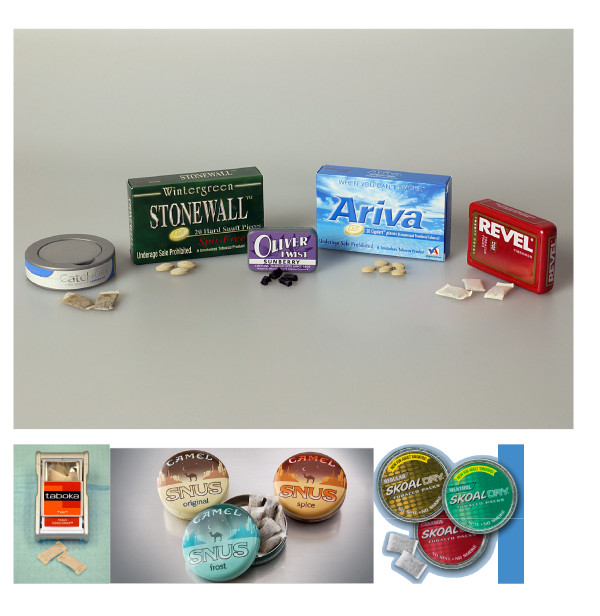
Modern smokeless tobacco products.

Over the past few years several ST products have emerged that are not easily classified into one of the previous groups. In fact, one reason for the popularity of moist snuff is that manufacturers have gradually refined the products in this category to be more user-friendly. The traditional pinch of moist snuff is difficult to keep in place, and the resultant migration is esthetically displeasing. Modern moist snuff products are sold in pre-portioned pouches similar to teabags, but much smaller. Because these products remain stationary in the mouth and generate very little juice, they can be used discreetly with no expectoration. There is a recent trend among manufacturers to offer even smaller pouches that are dry, with a wide range of non-tobacco flavors. Other products in this category consist of small pieces of leaf tobacco and pellets of compressed tobacco that dissolve completely. These products all share one important characteristic: they are of sufficiently small size that they can be used invisibly, and without expectoration.

### C. Prevalence

The prevalence of ST use has not received nearly as much attention as that of smoking, but adult prevalence has been documented by the National Health Interview Survey (NHIS). For adults, NHIS defines current ST users as those individuals who have used ST at least 20 times in their lives and are using ST every day or some days. In 1991 the prevalence of current ST use among adult men in the U.S. was about 5.6% (4.8 million), which declined to 4.4% (4.4 million) in 2000. In 1991 about 0.6% (533,000) of adult women in the U.S. were current users, and prevalence declined to 0.3% (324,000) by 2000 [42,43].

In 2000 the prevalence of ST use was higher among men age 18–44 years (6%) than among those age 45+ years (3%). Men in the Southern U.S. had the highest prevalence (7%) and those in the Northeast had the lowest (2%). As with smoking, prevalence of ST use was higher among men with a high school education or less. Finally, higher male prevalence was seen in rural areas (9%), compared with urban areas (3%) [43].

In the U.S. the number of male smokers is ten-fold higher than the number of ST users, so it follows that concurrent use of both products is common among ST users, but rare among smokers. About 25% of men who use ST report concurrent smoking, whereas concurrent use occurs in fewer than 5% of men who smoke [44]. Cigarette consumption is considerably lower in combined users compared with exclusive smokers [45-47].

### D. Health effects

#### 1. Oral leukoplakia

Oral leukoplakia is an ominous sounding term used frequently in discussions about ST use. The term literally means "white plaque," and it is used to describe areas of the mouth lining that become thickened by ST use or smoking. The World Health Organization has determined that leukoplakias resulting from ST use are considerably different from those resulting from smoking. The distinctions are based on the frequency of occurrence, the location in the mouth, and how often these leukoplakias result in mouth cancer [48,49].

The condition is rare, occurring in less than 1% of the general population, primarily in long-time smokers 40 to 60 years old [50,51]. Smoking-related leukoplakias most commonly involve the undersurface of the tongue and throat area, locations that account for 75% of oral cancer in the U.S. [51,52].

Oral leukoplakias occur in up to 60% of ST users [53,54], within 6 months to 3 years of starting ST use [55,56]. They primarily occur at the site of ST use and are largely a result of local irritation [55,57]. The frequency of appearance depends on the type of ST that is used. Moist snuff, which is more alkaline than chewing tobacco, more often leads to leukoplakia [56]. However, moist snuff in pre-portioned pouches causes fewer cases of leukoplakia than does the loose form [58].

There are distinct differences in how often ST and smoking leukoplakias show pre-cancerous changes called dysplasia. Dysplasia is seen infrequently in ST leukoplakias (less than 3%) [49,59-61]. Furthermore, even when dysplasia is present in ST leukoplakia, it usually is found in earlier stages than in leukoplakias due to smoking [62,63], where it is seen in about 20% of cases [64].

ST leukoplakias only rarely progress to cancer. For example, one prospective study found no case of cancer in 1,550 ST users with leukoplakia who were followed for 10 years [65], and a second study reported no case of oral cancer among 500 regular ST users followed for six years [66]. A retrospective study of 200,000 male snuff users in Sweden found only one case of oral cancer per year, an extremely low frequency [67]. In comparison, a follow-up study reported that 17% of smoking leukoplakias transformed into cancer within seven years [68].

In conclusion, oral leukoplakia occurs commonly in ST users, but it primarily represents irritation and only very rarely progresses to oral cancer.

#### 2. Oral cancer

ST use has been associated with oral cancer for many decades. It is widely perceived – both by laypersons and medical professionals – that the association is strong and applies to all ST products. However, epidemiologic studies dating back to the 1950s provide convincing evidence that most ST products increase oral cancer risks only minimally.

Rodu and Cole reviewed 21 epidemiologic studies published from 1957 to 1998 [69]. Unlike previous reviewers, these authors derived relative risk (RR) estimates for cancers of the mouth and associated upper respiratory sites related to use of chewing tobacco, moist snuff, dry snuff and a fourth category in which the type of ST was unclear or undetermined (ST unspecified). This study found that use of chewing tobacco and moist snuff were associated with only minimally elevated risks, while use of dry snuff conferred somewhat higher risks.

Chewing tobacco has been studied at least once in each of four decades from the 1960s to the 1990s. The data clearly show that chewing tobacco use is associated with only slightly elevated cancer risks; RRs for all anatomic sites are under 2 with confidence intervals including 1 (i.e. the risk elevation was not statistically significant) (Table 1). The first study evaluating the risk of chewing tobacco appeared in 1962 [70]. There were two studies in 1977 [71,72], two in 1988 [73,74], and four studies from 1993 to 1998 [75-78].

**Table 1:** Chewing Tobacco and Cancer of the Mouth and Upper Respiratory Sites

Anatomic Site	RR (95%CI)	Studies	Cases/Controls
Oral cavity	0.6 (0.3–1.3)	2	283/296
Pharynx	----		---
Oral cavity + pharynx	1.1 (0.8–1.6)	4	2113/4454
Larynx	1.3 (0.9–1.8)	1	387/2560
Oral + pharynx + larynx	1.7 (1.2–2.4)	2	362/457
All sites	1.2 (1.0–1.4)	8	3145/5245

As with chewing tobacco, summary RRs are only slightly elevated for moist snuff, with three RRs at or below 1 and the highest RR at 1.2 (Table 2). RRs for moist snuff were reported first in 1977 [71]. Another study appeared in 1988 [74], and five additional studies were published from 1993 to 1998, as this ST type came under intense scrutiny [75-79].

**Table 2:** Moist Snuff and Cancer of the Mouth and Upper Respiratory Sites

Anatomic Site	RR (95%CI)	Studies	Cases/Controls
Oral cavity	1.1 (0.8–1.6)	2	482/995
Pharynx	0.7 (0.4–1.4)	1	138/641
Oral cavity + pharynx	0.7 (0.4–1.2)	3	1682/3931
Larynx	1.2 (0.9–1.7)	2	544/3201
Oral + pharynx + larynx	---		---
All sites	1.0 (0.8–1.2)	5	2846/4926

Two of the seven studies on moist snuff were Swedish, both appearing in 1998 [78,79]. These studies have received considerable attention among tobacco researchers, particularly in Europe, because they are viewed as showing no oral cancer risk for Swedish products. They formed the basis for the Swedish government's decision in 1999 to recommend that the European Union (EU) oral cancer warning labels be removed from ST products. An EU directive in 2001 accomplished that objective and specified a new warning, "This tobacco product can damage your health and is addictive" [80]. Notably, the other five studies contributing to the summary RRs for moist snuff were American, and they reported RRs very similar to those of the Swedish studies.

Summary RRs for dry snuff use are higher, ranging from 4 to 13, although the confidence intervals for these estimates are wide (Table 3). The first study appeared in 1962 [70], followed by studies in 1981 [81], 1988 [73], and 1994 [76], spanning a period of 32 years.

**Table 3:** Dry Snuff and Cancer of the Mouth and Upper Respiratory Sites

Anatomic Site	RR (95%CI)	Studies	Cases/Controls
Oral cavity	----		----
Pharynx	----		----
Oral cavity + pharynx	4.0 (2.7–5.9)	3	298/947
Larynx	----		----
Oral + pharynx + larynx	13 (8.0–20)	1	93/393
All sites	5.9 (1.7–20)	4	391/1340

RRs for ST-unspecified range from 1.5 to 2.8, and most are statistically significant. For all sites the summary RR is 1.9 (CI = 1.5–2.3), which is intermediate between the low risks reported for chewing tobacco (1.2, 1.0–1.4) or moist snuff (1.0, 0.8–1.2) and the higher risk for dry snuff (5.9, 1.7–20) (Table 4). The intermediate risks for this ST category probably reflect the use of either the lower- or higher-risk products among different groups within the studies. Eight studies provided RRs for ST-unspecified, five of which appeared between 1957 and 1969 [82-86]. Additional studies appeared in 1992 [87], 1993 [75] and 1998 [88].

**Table 4:** ST-Unspecified and Cancer of the Mouth and Upper Respiratory Sites

Anatomic Site	RR (95%CI)	Studies	Cases/Controls
Oral cavity	2.8 (1.9–4.1)	4	581/798
Pharynx	2.3 (1.2–4.4)	3	169/472
Oral cavity +pharynx	1.5 (1.1–2.0)	3	655/2718
Larynx	1.8 (0.3–9.3)	1	23/100
Oral+pharynx+larynx	----		----
All sites	1.9 (1.5–2.3)	7	1428/3681

Tables 1 to 4 are adapted from [69].

Prior to the 2002 analysis by Rodu and Cole, the distinctive risk profiles of moist snuff and chewing tobacco on one hand, and dry snuff on the other, had gone unnoticed. In fact, the low oral cancer risk associated with chewing tobacco had been discussed briefly in only one article [89]. No distinction in risks had been made previously between dry snuff and moist snuff, even though these products are considerably different with regard to tobacco content and processing, as noted earlier.

The majority of epidemiologic studies regarding ST and oral cancer have limitations, many of which are typical for case-control studies, and some important for understanding unique oral cancer risks. Most of them did not control for confounding by two strong determinants of oral cancer, cigarette smoking and alcohol use. Positive confounding by smoking would occur if ST users smoke more than do nonusers of ST. This would result in artificially high risk estimates for oral cancer among ST users. On the other hand, negative confounding is plausible and would occur if smoking rates are lower among ST users than among nonusers of ST. This would result in artificially low risks for oral cancer among ST users.

Only three studies [78,79,81] controlled for alcohol use, where only positive confounding is likely. Thus, control for alcohol consumption in all studies probably would have reduced somewhat many of the estimates of mouth cancer risk associated with ST use.

However, even with these limitations, the results of these studies are reasonably consistent with regard to mouth cancer risks from long-term use of moist snuff and chewing tobacco. In their review Rodu and Cole concluded that "the abundance of data now available indicates that commonly used ST products increase the risk of oral and upper respiratory tract cancers only minimally."

Since the 2002 review four epidemiologic studies, one from Sweden and three from the U.S., have been published [90-93]. In all of these studies ST use was not associated with a significant increase in mouth cancer risk. In 2004 a group of epidemiologists concluded that the evidence linking ST use and oral cancer was "not decisive" [94]. These investigators commented that many claims in the media "overemphasize the risk of oral cavity cancer [from ST use], reaching beyond the scientific data."

In 2005 the American Cancer Society (ACS) reported that ST users did not have significantly increased risks for oral and pharyngeal cancer in either the first or the second Cancer Prevention Study [92]. Despite this finding, the ACS website continues to focus on ST as a cause of mouth cancer, erroneously stating that "risk of cancer of the cheek and gums may increase nearly 50-fold among long-term snuff users" [95]. A later section of this report will discuss this type of misinformation.

#### 3. Other cancers

As noted above, cigarette smoking is associated with increased risk for several cancers in locations not in contact with cigarette smoke. In comparison, numerous epidemiologic studies have not demonstrated that ST use is associated with risk of cancer at any site outside the mouth. In 2004 Waterbor et al. assessed the epidemiologic research literature and summarized the evidence regarding ST use and cancers in various locations [94]. Table 5 shows the conclusions of Waterbor et al. with respect to cancer risks associated with ST use, compared with the established risks for smoking.

**Table 5:** Risk of Cancer in Various Sites Associated with ST Use and Smoking

Cancer Site	Risks from ST Use*	Risks from Smoking**
Pharynx	No relationship	RR= 5–11
Larynx	No relationship	13–15
Lung	Inadequate	13–23
Stomach	Not persuasive	1.4–2.0
Kidney	No association	1.3–3.7
Esophagus	Not persuasive	7–8
Pancreatic cancer	Inconclusive	2.3
Bladder cancer	None	2.2–3.3

* From [94].

** Among current smokers (men and women), used by the CDC for national estimates of smoking-attributable mortality [96].

#### 4. Cardiovascular diseases

Over the past 15 years, eight epidemiologic studies have examined the risk of cardiovascular diseases among ST users. Six of the studies found that ST users had no increased risk for heart attacks or strokes [47,90,97-100]. The other two reported modestly positive associations, with ST users having RRs of 1.2 and 1.4 [92,101], which are lower than those of smokers. In 2003, Asplund completed a comprehensive review of the cardiovascular effects of ST use [102]. He concluded that, in distinct contrast to smokers, ST users do not exhibit any significant differences from nonusers of tobacco with regard to the following measures of cardiovascular health: heart rate, blood pressure, cardiac output and maximal working capacity, levels of hemoglobin and hematocrit, leukocytes, antioxidant vitamins, fibrinogen, components of the fibrinolytic system, C-reactive protein and thromboxane A2 production. In addition, ST users did not show important smoking-associated vascular changes, including increased thickness of blood vessels and atherosclerotic plaque development. In summary, most of the medical and epidemiologic evidence documents that ST users do not have elevated risks for cardiovascular diseases.

Two studies based in Sweden have examined the impact of ST use as a risk factor for adult-onset diabetes. One of these studies found that current ST users had a slightly elevated risk (Odds ratio = 1.5, CI = 0.8–30) [103], while the other reported that the risk of diabetes in ST users was not significantly increased [104].

## IV. Scientific rationale for harm reduction with ST

### A. Nicotine maintenance

#### 1. Nicotine background

Nicotine has been characterized as powerfully addictive. But nicotine itself poses little or no health hazard. For example, it does not cause emphysema or cancer [105,106], and there is no evidence that it plays a direct role in the development of cardiovascular diseases [106,107]. A report from a meeting at the United Nations Focal Point on Tobacco or Health concluded that "long-term nicotine use is not of demonstrated harm, with the possible exception of use during pregnancy" [108].

The U.S. Food and Drug Administration (FDA) has acknowledged the safety of nicotine replacement therapy (NRT) by allowing its sale without prescription. Long-term use of NRT has not been associated with any medical risks and is considered far less hazardous than relapsing to smoking cigarettes [109,110], prompting authorities in the United Kingdom (U.K.) to liberalize NRT regulations there recently. The new guidelines allow NRT use by patients with cardiovascular disease, by confirmed smokers ages 12 to 17, by pregnant smokers, and concurrently by those who continue to smoke [111].

Nicotine gum was introduced in the U.S. in 1984 as a prescription product to assist in smoking cessation . The gum is considered to pose no consequential health hazard, and it was granted over-the-counter status by the FDA in 1996. The gum gives the user only a limited degree of control over the amount of nicotine absorbed because its nicotine content is low and only slowly released [112]. Depending on state and local excise taxes and cigarette consumption, the gum may be competitive on a per-unit basis for the smoker. However, it is available only in large quantities, making the purchase price far more expensive than that for cigarettes, a major economic disincentive. In fact, cost is the reason most frequently cited by smokers for never using NRT [113].

The nicotine patch was introduced in the U.S. in 1992 and was available without prescription by 1996. It continuously delivers nicotine through the skin for up to 24 hours. Although the patch is intended to preclude smoking, the rate of nicotine delivery is so low that smoking while wearing the patch is not uncommon. The patch's major limitation is its inadequate nicotine delivery, but it is not a technical problem. A high-dose nicotine patch has been evaluated and may provide complete nicotine replacement even for heavy smokers [114].

Many smokers overestimate the health risks of NRT products. A 2001 survey of 1,046 adult smokers found that 53% incorrectly believed nicotine causes cancer and 14% didn't know [115], and a 2002 survey found that half of all smokers are concerned about negative side effects of using NRT [116]. A similar problem exists in the U.K., where recent research found that 69% of smokers believe NRT is as harmful as cigarettes.

Misconceptions are not limited to persons without medical training. Twenty-two percent of general medical practitioners in the U.K. are concerned that NRT is just as harmful as cigarettes, 40% believe that nicotine may cause cardiovascular disease and stroke, and one-quarter believe it may cause lung cancer [117].

In summary, poor nicotine delivery, high cost and misconceptions about health risks are the principal reasons that the long-term quit rate among users non-prescription nicotine medications is only 7%, according to a recent meta-analysis [118].

#### 2. Long-term use of nicotine medications

The FDA specifies that nicotine medications should not be used for more than 10 to 12 weeks. This restriction is based not on health considerations, but on a concern about prolonging nicotine addiction. Considering the limitations of nicotine medications, it is remarkable that some smokers continue to use the products beyond the 3-month period specified by the FDA. About 20 percent of those who quit smoking with nicotine gum used it for more than one year when it was available only by prescription [112]. A cessation study that provided free gum but encouraged weaning after two months use reported that 37% of smoke-free subjects at one year were still using nicotine gum [119]. Using a liberal definition of continuous use, a recent study found that as many as one-third of current nicotine gum users have used the product for longer than six months [120]. That study also reported that, among persons who start to use nicotine gum, 7% will use it for longer than six months and 1% will continue use for over two years. The equivalent figures for nicotine patch were 1.7% and 0.05% respectively.

#### 3. Nicotine concentration and availability from ST products

ST products contain nicotine at far higher concentrations than nicotine medications, and at levels that are generally acknowledged to be addictive [121,122]. Bioavailability of nicotine from ST products is dependent on the pH of the product, since unprotonated nicotine (in more alkaline products) is absorbed more efficiently and more rapidly across the mucous membranes of the mouth than protonated forms of the drug from more acidic products. The pH-dependent absorption kinetics of nicotine is a very important reason why ST is not consumed like foods. The pH of stomach contents is very acidic, which strongly inhibits the absorption of nicotine [122].

The nicotine absorption profiles of ST products, which have been known for many years [105,123], show both advantages and disadvantages when compared with those from smoking. Nicotine absorption from ST is somewhat slower than that from cigarettes, although the peak nicotine levels obtained in venous blood are similar [105]. In addition, elevated serum nicotine from ST use persists for much longer than that from smoking [105]. This may explain the observation that unit consumption of ST products among former smokers was much lower than prior unit consumption of cigarettes [124,125]. In the end, ST users and smokers consume similar quantities of nicotine daily [126].

### B. Comparison of risks from ST use and smoking

The established health risks associated with ST use are vastly lower than those of smoking. In the past 25 years, almost 80 peer-reviewed scientific and medical publications have acknowledged the differential risks between the two tobacco products (see Additional File 1).

In 1980 Michael A.H. Russell and co-workers proposed that powdered nasal snuff might serve as an effective substitute for cigarettes because it delivers nicotine effectively without the risks of tobacco combustion [4]. This article was cited shortly thereafter in a brief letter in the *New England Journal of Medicine *[5]. Russell et al. published follow-up studies on nasal snuff in 1981 [6] and on an oral ST product in 1985 [7]. Lynn Kozlowski, a prominent American smoking and nicotine addiction expert at Penn State University, noted in 1984 and 1989 that smokeless forms of tobacco conferred fewer risks to users and therefore might serve as effective substitutes for cigarettes [8,9,127]. Starting in 1994, University of Alabama at Birmingham researchers Brad Rodu and Philip Cole provided a quantitative assessment of the difference in risks for the two products. Using established risk estimates from accepted sources, Rodu and Cole documented that ST use confers only about 2% of the health risks of smoking [10-12]. In addition, they established that the average reduction in life expectancy from long-term ST use was about 15 days, compared with a reduction of about 8 years from smoking [11].

In 1994 Rodu noted that ST use posed a lower risk for mouth cancer than smoking [10]. In 2001 this was confirmed by a comprehensive report on tobacco harm reduction by the Institute of Medicine, which stated that "the overall [oral cancer] risk [for ST use] is lower than for cigarette smoking, and some products such as Swedish snus may have no increased risk" [15].

By the late 1990s some influential organizations acknowledged the differential risks of ST use and smoking. For example, in 1997 experts meeting at the United Nations Focal Point on Tobacco or Health concluded that "it is now evident that the risk of death and disease is related to not only the amount but also the nature of tobacco exposure; for example, daily cigarette smoking is far more dangerous than occasional use of Swedish snuff" [108]. That same year a scientific panel convened by the Swedish National Board of Health and Welfare concluded that "the health risks related to smokeless tobacco are with great probability lower than those related to smoking" [41].

In 2002 the Royal College of Physicians of London, one of the oldest and most prestigious medical societies in the world, issued a report called "Protecting Smokers, Saving Lives," which stated, "As a way of using nicotine, the consumption of non-combustible [smokeless] tobacco is on the order of 10–1,000 times less hazardous than smoking, depending on the product." The report continued with an even bolder statement, acknowledging that some smokeless tobacco manufacturers may want to market their products "as a 'harm reduction' option for nicotine users, and they may find support for that in the public health community" [128]

In 2004 a study funded by the NCI assembled an international panel of experts (including epidemiologists from the NIH and the ACS) to compare the risks of ST use with those of smoking. The study authors reported that, "In comparison with smoking, experts perceive at least a 90% reduction in the relative risk of low-nitrosamine smokeless tobacco use." The authors concluded that "This finding raises ethical questions concerning whether it is inappropriate and misleading for government officials or public health experts to characterize smokeless tobacco products as comparably dangerous with cigarette smoking" [129].

Phillips et al. have provided perhaps the most detailed and direct comparison of risks from use of Swedish or American ST products and from smoking, using a spectrum of risk estimates for ST use ranging from well-substantiated and plausible to highly speculative and implausible [130]. They estimated that, compared with smoking, ST risks "in the range of 1% or 2%, and possibly less, are most consistent with the epidemiologic evidence. Perhaps most important, our calculation shows that comparative risk estimates as high as 5%, let alone 10% or more, cannot be justified based on the evidence."

### C. Evidence that ST is an effective substitute for cigarettes

#### 1. Survey data

There is limited evidence from governmental and other surveys that some smokers have quit by substituting ST products for cigarettes, and most of the published information on this subject is dated. The 1991 NHIS survey revealed that 33.3% (about 1.8 million) of adult current ST users were former cigarette smokers [42].

The 1986 national Adult Use of Tobacco Survey, conducted by the CDC Office on Smoking and Health, found that 7% (1.7 million) of male ex-smokers had used ST to help them quit smoking cigarettes. That same survey found that only 1.7% of male ex-smokers (404,600) had used organized programs to help them quit smoking [131].

The 1998 NHIS survey revealed that 5.8% of daily snuff users reported quitting smoking cigarettes within the past year, that daily snuff users were three times more likely to report being former cigarette smokers than never snuff users, and that daily snuff users were four times more likely to have quit smoking in the past year than never snuff users [132].

According to the 1987 NHIS survey, 23- to 34-year old U.S. men who had smoked cigarettes and subsequently used snuff were twice as likely to have quit smoking (95% CI 1.2 – 3.5) than were cigarette-only users [133].

Cohen-Smith and Severson surveyed 51 female and 59 male ST users in the Northwestern U.S., 98% and 90% of whom respectively were either current or former cigarette smokers. They found that 52% of women and 59% of men used ST in place of cigarettes while quitting smoking [134].

#### 2. Clinical trial data

One clinical trial, an open-label, nonrandomized pilot study, has been conducted assessing the efficacy of an ST product in helping cigarette smokers become smoke-free. The investigators used a low-intensity approach, consisting of a 20-minute lecture about the health effects of all forms of tobacco use, followed by information about and samples of pre-portioned single-dose tobacco packets available throughout the U.S. The investigators used exhaled carbon monoxide levels to validate participant self-reports regarding smoke-free status at the conclusion of the original study after one year [125] and after seven years of follow-up [135].

Of 63 subjects starting the study, 16 had successfully quit smoking by switching to ST after one year, and 12 were still smoke-free after seven years. At enrollment, the average cigarette consumption of the successful participants had been 1.5 packs per day. One year later average consumption of ST was 2.3 packages per week among the 13 successful quitters using ST (3 were tobacco-free). Four additional participants had used ST to reduce their cigarette consumption by at least 50%.

#### 3. The Swedish tobacco experience

For the past 100 years, cigarette smoking has been the dominant form of tobacco consumption in almost all developed countries. One notable exception is Sweden, where smoking rates, especially among men, have been considerably lower than those of comparable countries for decades. (An ACSH article provides historical background on Swedish snus [136]). Over the past 50 years Swedish men have had the lowest rates of smoking-related cancers of the lung, larynx, mouth and bladder in Europe [137], and the lowest percentage of male deaths related to smoking of all developed countries [138,139].

A 2004 study revealed that if men in the (15-country) EU had the smoking prevalence of Sweden, almost 200,000 deaths attributable to smoking would be avoided each year [140]. In contrast, women in Sweden smoke at rates much more similar to women in other European countries, and this is reflected in similar rates of smoking-related illnesses. The 2004 study found that only 1,100 deaths would be avoided in the EU at Swedish women's smoking rates.

As Fagerström pointed out in a recent study, per capita consumption of nicotine from tobacco in Sweden is quite high and on par with other countries such as Denmark, the U.S. and Austria [141]. The difference between Sweden and the other countries is how nicotine is consumed. In Denmark, the U.S. and Austria, almost all nicotine consumption is derived from tobacco combustion. In contrast, ST use, in the form of snus, accounts for almost 50% of all contemporary nicotine consumption in Sweden. Snus use in Sweden is much more common among men than among women; over 60% of nicotine consumption among Swedish men is from snus. This is not a new phenomenon; for over a century, Swedish men have had among the world's highest per capita consumption of ST [142].

Beginning in 2002, an American-Swedish research group used a World Health Organization database to describe in detail the impact of snus use on smoking among the population in northern Sweden during the period 1986–2004 [46,143,144].

Among men, the prevalence of all tobacco use was stable during the study period, at about 40%. However, there were striking, and opposite, changes in prevalence of smoking and snus use. Smoking prevalence was 19% in 1986, and it was lower in all subsequent surveys, reaching 9% in 2004. The prevalence of exclusive snus use increased from 18% in 1986 to 27% by 2004. Snus use was the dominant factor in the higher prevalence of ex-smoking among men compared to women (prevalence ratio 6.18, 95% CI 4.96 – 7.70).

Among women the prevalence of all tobacco use also was steady at 27 to 28%, and women smoked at higher rates than men in all surveys. But these studies showed that snus use was associated with lower smoking rates among women in 1999 and 2004. Smoking prevalence was about 25 to 27% in 1986, 1990 and 1994, but declined to 21% in 1999, and 16% in 2004. The prevalence of snus use was 0.5% in 1986 and increased to 1.9% in 1990, 2.0% in 1994, 5.1% in 1999 and 8.9% in 2004.

In these reports snus use was not associated with smoking initiation, as the prevalence of smoking among former snus users was low in all survey years (3–4%). The evidence showed that among adult men in northern Sweden the dominant transition is from smoking to snus, not vice versa.

In 2003 Foulds et al. reviewed the evidence relating to the effects of snus use on smoking and concluded, "Snus availability in Sweden appears to have contributed to the unusually low rates of smoking among Swedish men by helping them transfer to a notably less harmful form of nicotine dependence." The investigators noted that "in Sweden we have a concrete example in which availability of a less harmful tobacco product has probably worked to produce a net improvement in health in that country" [145].

In 2005 Furberg et al examined tobacco use data from the Swedish Twin Registry, finding that regular snus use was associated with smoking cessation, not initiation, among almost 15,000 male participants. Both regular and occasional snus use were protective against having ever smoked [146].

In 2006 Ramstrom and Foulds examined data from a 2001–02 nationally representative Swedish social survey. They found that snus use among men was significantly protective against smoking initiation (OR = 0.3, CI 0.2–0.4). They also found that snus was the most commonly used cessation aid among men (used by 24% of men on their most recent quit attempt). Men who used snus as a quit-smoking aid were more likely to quit successfully than those using nicotine gum (OR = 2.2, CI = 1.3–3.7) or the patch (OR = 4.2, CI = 2.1–8.6), which was also true for women [147].

## V. Policy issues

### A. ST use: gateway to smoking cessation, not smoking initiation

Data from research studies in Sweden and the U.S. do not support the allegation that widespread use of ST serves as a gateway to smoking, especially among youth. A 2003 policy statement published in *Tobacco Control*, coauthored by Clive Bates, former director of Action on Smoking and Health (U.K.) and five other eminent tobacco research and policy experts, dismissed the notion that ST use led to smoking in Sweden: "To the extent there is a 'gateway' it appears not to lead to smoking, but away from it and is an important reason why Sweden has the lowest rates of tobacco related disease in Europe" [148]. Foulds reached a similar conclusion: "This review suggests...that in Sweden snus has served as a pathway from smoking, rather than a gateway to smoking among Swedish men" [145].

A 2005 study examined tobacco use among 15- to 16-year old schoolchildren over a 15-year period, from 1989 to 2003 [149]. The investigators found that the prevalence of regular snus use among Swedish boys increased from about 10% to 13% from 1989 to 2003, but the prevalence of regular smoking was very low and declined, from about 10% to under 4%. The prevalence of snus use among girls was very low, and the prevalence of smoking was about double that of boys over the entire period. The authors concluded that snus use did not appear to be a gateway to smoking among Swedish youth but instead was associated with low smoking prevalence among boys.

In the U.S. investigators have not found credible evidence that ST use is a gateway to smoking among American youth. In 2003 Kozlowski et al analyzed data from the 1987 NHIS survey and concluded that there was little evidence that ST use was a gateway to smoking, because the majority of ST users had never smoked or had smoked cigarettes prior to using ST [133]. The investigators noted that their results coincided with earlier work from Sweden and with a tobacco industry-sponsored survey from 1984 [150].

In 2003 O'Connor et al. examined data from the 2000 National Household Survey on Drug Abuse [151]. They described the impact of ST use on subsequent cigarette smoking initiation as "minimal at best." O'Connor et al. also examined data from the CDC's Teenage Attitudes and Practices Survey for evidence that ST use served as a gateway to smoking among youth [152]. They concluded that ST use was not associated with smoking initiation after appropriate control for confounding by well-recognized psychosocial predictors of smoking. This is in contrast to an earlier report that did not control for confounding and found a positive association [153].

Claims of a gateway effect persist, even with lack of credible evidence, prompting O'Connor et al. to note in 2005, "Continued evasion of the [harm reduction] issue based on claims that ST can cause smoking seems, to us, to be an unethical violation of the human right to honest, health-relevant information" [154]. That quote introduces the next topic, information and misinformation about ST and tobacco harm reduction.

### B. Information and misinformation about ST and tobacco harm reduction

Kozlowski et al. have argued persuasively that smokers have a fundamental right to accurate information about safer forms of tobacco use [155-157]. The research group established the underlying rationale for the provision of this information, citing principles of the Universal Declaration of Human Rights, the doctrine of informed consent, and business ethics contract theory, under which companies have a moral obligation to inform customers about important information regarding their products.

In 2001 the U.S. Supreme Court may have provided a legal basis for holding tobacco manufacturers responsible for providing truthful information about the differential risks of ST use and smoking. Writing the majority opinion in Lorillard v. Reilly, in which a 5–4 majority of the Court ruled that broad advertising restrictions by the Commonwealth of Massachusetts violated the commercial free-speech rights of tobacco manufacturers, Justice Sandra Day O'Connor wrote that "the State's interest in preventing underage tobacco use is substantial, and even compelling, but it is no less true that the sale and use of tobacco products by adults is a legal activity. We must consider that tobacco retailers and manufacturers have an interest in conveying truthful information about their products to adults, and adults have a corresponding interest in receiving truthful information about tobacco products" [158].

#### 1. Fundamental right to information

Over the past 20 years, many public health and tobacco policy experts have argued that smokers have a fundamental right to accurate information about less hazardous products so that they can make informed choices if they are unable or unwilling to quit tobacco altogether. In 1984 Kozlowski commented on both the challenges and the potential of tobacco harm reduction, writing that "the use of less-hazardous tobacco, if prohibitionist impulses can be put aside, may have an important role in the treatment of the smoking and health problem..." [9].

In 1994 Rodu proposed that a "public health policy that recognizes ST as an alternative to smoking would benefit individuals confronted with the unsatisfactory options of abstinence or continuing to smoke" [10]. In a 1995 book, Rodu told smokers that "ST products allow you, the hard-core and long-term smoker, to take back a measure of control over your health by indulging in a far safer form of tobacco use" [13].

One concern about tobacco harm reduction is that dissemination of information about less hazardous tobacco products might adversely affect public health if it creates new users. However, the risk/use equilibrium addresses this issue [159]. If ST use is 50 to 100 times less hazardous than smoking, it would require 50 to 100 more ST users to reach the level of public harm produced by smoking. In other words, it would take 2.3 to 4.5 billion ST users to have the same death toll as 45 million American smokers do today, an impossible scenario in the U.S. population of 290 million people.

Kozlowski's message in 2002 was clear: "Cigarettes kill about half of those who smoke them...It is urgent to inform smokers about options they have to reduce risk...public health policy in this instance lacks compelling justification to override the human rights of the individual. Individuals have the right to such relevant information [on tobacco risks]" [155]. That same year, the prestigious Royal College of Physicians of London made its hopeful statement that "some manufacturers may want to market ST as a 'harm reduction' option for nicotine users, and they may find support for that in the public health community" [128].

Since then a growing number of experts have weighed in on the case for providing smokers relevant risk information and safer tobacco options. In 2002 Cummings argued for a market approach involving risk information: "Until smokers are given enough information to allow them to choose products because of lower health risks, then the status quo will remain. Capitalism, and not government regulation, has the greatest potential to alter the world-wide epidemic of tobacco-related disease" [160].

In 2003 Kozlowski et al expanded on the rationale that smokers are entitled to information about safer products, addressing concerns that provision of risk information might adversely affect public health: "Public health concerns should trump individual rights only when there is clear and convincing evidence of harm to society. Lacking that evidence, individual rights should prevail" [161].

#### 2. Misinformation from governmental and other organizations

Americans are badly misinformed about the risks of ST use, especially in comparison with smoking. In 2005 a survey of 2,028 adult U.S. smokers found that only 10.7% correctly believed that ST products are less hazardous than cigarettes [154]. In another survey, 82% of U.S. smokers incorrectly believed that chewing tobacco is just as likely to cause cancer as smoking cigarettes [162].

A 1999–2000 survey of 36,012 young adults entering the U.S. Air Force found that 75% of males and 81% of females incorrectly believed that switching from cigarettes to ST would not result in any risk reduction, while another 16% of males and 13% of females incorrectly believed that only a small risk reduction would occur. Only 2% of males and 1% of females correctly understood that a large risk reduction would occur by switching from cigarettes to ST [163]. That survey also found that the overwhelming majority of subjects believed that switching from regular to low-tar cigarettes conferred greater reduction in risks than switching from cigarettes to ST.

It is not clear how Americans have become so confused about tobacco risks. But it is clear that misinformation about ST products is available in copious quantities from ostensibly reputable sources, including governmental health agencies and health-oriented organizations. Phillips et al have made some of the most pointed comments about this phenomenon:

"Certain health advocates believe it is acceptable to mislead people into making choices they would not otherwise make...Through the use of various tactics, advocates who oppose the use of ST as a harm reduction tool have managed to convince most people that the health risk from ST is several orders of magnitude greater than it really is. The primary tactic they use is making false or misleading scientific claims that suggest that all tobacco use is the same. . . . Apparently motivated by their hatred of all things tobacco, they are trying to convince people to not switch from an extremely unhealthy behavior to an alternative behavior that eliminates almost all of their risk" [164].

The tactic has worked in the U.S., as Americans, almost without exception and regardless of general and health education levels, believe that the risks from ST are similar to those from smoking. In particular, Americans incorrectly believe that switching from smoking to ST use will create a large increased risk for oral cancer. Phillips has characterized this popular misinformation as the "you might as well smoke" message, since it tells people that if they are using ST, they could switch to smoking with no increase in risk, while smokers considering switching to ST should not bother [165].

Phillips et al. systematically reviewed content about ST use on the web in 2003 and found that the risks of ST use are almost always conflated with those of smoking [165]. Roughly one-third of the time, there are explicit claims that ST is as bad as or worse than smoking. Most of the rest of the time the information is arranged to imply similar risks, though there is no such explicit statement. There are also a variety of specific claims that are not supported by the literature.

Government agencies, other organizations and members of the public health community have a moral obligation **not **to misinform smokers about products that have fewer risks than cigarettes. Nevertheless, researchers have exposed numerous cases of misinformation from governmental sources. For example, in 2003 Kozlowski and O'Connor criticized websites of the CDC and the Substance Abuse and Mental Health Services Administration for erroneously reporting that ST products were not safer than cigarettes, pointing out that "the misleading health information on ST fails to meet the government criteria against deception in research" [156].

At a 2003 U.S. House subcommittee hearing, U.S. Surgeon General Richard Carmona testified: "I cannot conclude that the use of any tobacco product is a safer alternative to smoking...There is no significant evidence that suggests ST is a safer alternative to cigarettes" [166]. Scott Leischow, Chief of the Tobacco Control Research Branch at the NCI, presented similar testimony at a concurrent hearing [167]. Carmona's statement prompted Rodu, who also presented testimony at that hearing [168], to comment that the Surgeon General was "sadly ill-informed about the nation's No. 1 health problem, cigarette smoking." Rodu strongly criticized Carmona, writing that he should be compelled to "tell American smokers the truth about all available options for quitting. After all, the 10 million smokers who will die over the next two decades are, in a very tangible way, his responsibility and his legacy" [169].

In March 2004, Ken Boehm of the National Legal & Policy Center (NLPC), a non-profit organization committed to promoting open, accountable and ethical practices in government, filed a request under the Data Quality Act (DQA) for correction of a document from the National Institute of Aging (NIA) that contained misinformation regarding the relative risks of ST versus cigarettes. (This other DQA requests on ST can be seen at the U.S. Department of Health and Human Services website [170]) The request resulted in a change of wording from the original text:

"Some people think ST (chewing tobacco and snuff), pipes, and cigars are **safer **than cigarettes. They are not."

The revised wording from NIA was:

"Some people think ST (chewing tobacco and snuff), pipes, and cigars are **safe**. They are not."

The claim that ST products are not "**safe" **is a tactic that can be traced back to the 1986 Comprehensive Smokeless Tobacco Education Act, which required as one of three warnings on all ST products: "This product is not a safe alternative to cigarettes."

In 1995 Rodu criticized this warning as ludicrous and suggested that other consumer products like automobiles, lawnmowers, aspirin and red meat don't meet absolute criteria for safety [13]. A decade later, Kozlowski and Edwards criticized this type of uninformative warning in a study entitled, "'Not safe' is not enough: smokers have a right to know more than there is no safe tobacco product" [157]. These authors believe that smokers deserve more information: "The 'not safe' or 'not harmless' messages don't address the reality that some tobacco products are substantially safer than others... Saying tobacco 'isn't safe' isn't incorrect, but it isn't saying enough. Going beyond the no safe tobacco message to provide better information on the nature of risks from tobacco products and nicotine delivery systems is necessary to respect individual rights to health relevant information."

Ken Boehm from NLPC summarized the arguments against misinformation:

"This is the kind of evidence Americans should be able to review and make their own decisions. Despite the best efforts of the largest government bureaucracy in the history of the republic, Americans still prefer to do their own thinking. And as we do our own thinking on the merits of reduced-risk products such as ST, none of us needs misinformation supplied by our own government" [171].

With regard to a policy as "credible, logical and eminently do-able" as tobacco harm reduction [172], it is unfortunate that arguments against deception are actually necessary.

## VI. Conclusion and recommendations

The past 40 years have brought ever more assertive public health campaigns against cigarette smoking. A coalition of well-funded public and private agencies has as its goal a reduction in the prevalence of cigarette smoking. The coalition's influence has resulted in pervasive health warnings, ever more intensive quit-smoking programs, and recently the social ostracism of smokers and the industry that supplies them. Yet 45 million Americans continue to smoke, and far too many die from smoking-related diseases.

The American Council on Science and Health has been part of this anti-smoking coalition for several decades. Throughout its history ACSH has published many articles about the health risks of smoking. And it has held the tobacco industry accountable for its part of the devastating toll from tobacco. ACSH founder Elizabeth Whelan published a landmark anti-smoking book, *A Smoking Gun?: How the Tobacco Industry Gets Away with Murder *[173].

ACSH was founded in 1978 by a group of scientists who had become concerned that many important public policies related to health and the environment did not have a sound scientific basis. These scientists created the organization to add reason and balance to debates about public health issues and bring common-sense views to the public.

The mission of the ACSH is to promote sound science in regulation, in public policy, and in the courtroom and to assist consumers, via the media, in distinguishing real health threats from purely hypothetical ones. ACSH believes that strong support of tobacco harm reduction is fully consistent with this mission; as this report documents, there is a strong scientific and medical foundation for tobacco harm reduction, and it shows great potential as a public health strategy to help millions of smokers.

Tobacco harm reduction empowers smokers to gain control over the consequences of their nicotine addiction. At its simplest it is nonintrusive and solely educational, and therefore has a strong moral rationale. The strategy is cost-effective and accessible today to almost all smokers. But its implementation will require rethinking of conventional tobacco control policies and their premises.

The ACSH believes that the following actions will benefit smokers:

1. **Agencies of the federal government (most notably the Office of the Surgeon General) and health promotion organizations (such as the American Cancer Society and the Mayo Clinic) should discontinue the campaign of misinformation that irresponsibly misrepresents the scientific information about and use of ST products. **They endanger their reputations as sources of trusted health information by providing messages about ST products that are neither accurate nor credible. The campaign of misinformation should be replaced with an educational program that emphasizes the differential risks of all forms of tobacco use.

2. **Regulatory restrictions on the manufacture and sale of nicotine replacement medications should be revised. **Nicotine is addictive, but it plays little or no role in the development of most smoking-related diseases. Manufacturers of nicotine replacement medications should be permitted to sell higher doses of the drug within flavor/delivery systems that are satisfying and enjoyable for smokers at costs that are competitive with cigarettes. In addition, smokers should be informed that permanent use of NRT is vastly safer than continuing to smoke. This could be accomplished by new labels on NRT packaging and additional labels on cigarette packs: "Notice: Nicotine does not cause cancer, heart diseases or emphysema."

3. **Manufacturers of tobacco products should follow the lead of British American Tobacco (BAT) and acknowledge that ST use is vastly safer than smoking. **BAT has openly admitted that oral ST products are safer than cigarettes, and this company is actively engaged in test-marketing Swedish snus in Sweden, Norway and South Africa [174]. At the press date of this report, cigarette manufacturers in the U.S. have introduced ST products in limited test markets, but they have made no statements regarding differential health risks. This is unacceptable, given the state of the science documented in this report.

4. **Any federal legislation that addresses the regulation of tobacco should include provisions that adequately reflect the differences in risks between combustible tobacco products and ST products or NRT. **This includes careful review of current proposals before Congress to ensure that the legislation is written to regulate the labeling and marketing of products based on their risks. The goal should be to give users of tobacco the necessary information they need to understand the differences between various tobacco and nicotine products so they can make the appropriate health choices and decisions.

5. **Pending enactment of more comprehensive regulation, the U.S. Congress should repeal the federally-mandated warning that now appears on ST products: **"This product is not a safe alternative to cigarettes." This warning not only misleads smokers; it may send a message to ST users that they might as well smoke. The warning should be replaced with the following, which would appear as an onsert with cigarette packages – "Warning: Smokeless tobacco use has risks, but cigarette smoking is far more dangerous. Quitting tobacco entirely is ideal, but switching from cigarettes to ST can reduce greatly the health risks to smokers and those around them." Placement of this warning with cigarettes ensures that it reaches the target audience, continuing smokers.

6. **State legislatures should follow the lead of Kentucky and establish rational risk-based tax policies for tobacco products. **In 2005 the Commonwealth of Kentucky enacted an excise tax structure for cigarettes and ST products that was based on differential risks. The final bill stated: "The General Assembly recognizes that increasing taxes on tobacco products should reduce consumption, and therefore result in healthier lifestyles for Kentuckians. The relative taxes on tobacco products proposed in this section reflect the growing data from scientific studies suggesting that although smokeless tobacco poses some risks, those health risks are significantly less than the risks posed by other forms of tobacco products. Moreover, the General Assembly acknowledges that some in the public health community recognize that tobacco harm reduction should be a complementary public health strategy regarding tobacco products. Taxing tobacco products according to relative risk is a rational tax policy and may well serve the public health goal of reducing smoking-related mortality and morbidity and lowering health care costs associated with tobacco-related disease."

## Abbreviations

**ACS **American Cancer Society

**ACSH **American Council on Science and Health

**BAT **British American Tobacco

**CDC **Centers for Disease Control and Prevention

**DQA **Data Quality Act

**EU **European Union

**FDA **Food and Drug Administration

**NCI **National Cancer Institute

**NHIS **National Health Interview Survey

**NIA **National Institute on Aging

**NIH **National Institutes of Health

**NLPC **National Legal Policy Center

**NRT **Nicotine replacement therapy

**RR **Relative risk

**ST **Smokeless tobacco

**TSNA **Tobacco specific nitrosamine

**U.K. **United Kingdom

**U.S. **United States

## Competing interests

Dr. Rodu is supported by unrestricted grants from the US Smokeless Tobacco Company and Swedish Match AB to the University of Louisville. The sponsors are unaware of this work, and thus had no scientific input or other influence with respect to its design, analysis, interpretation or preparation of the manuscript. Dr. Rodu has no other financial or other personal conflict of interest with respect to tobacco use or cessation.

Mr. Godshall declares that he has no competing interests.

## Authors' contributions

Both authors participated in the literature review and drafting of the manuscript.

## Supplementary Material

Additional File 1 Peer-Reviewed Scientific/Medical Articles Acknowledging that Smokeless Tobacco Use Confers Less Risk Than Cigarette Smoking, In Reverse Chronological Order 2006-1980. Click here for file
