# Supplementary material for: Tobacco harm reduction: an alternative cessation strategy for inveterate smokers
Source: Harm Reduct J. 2006 Dec 21;3:37. doi: 10.1186/1477-7517-3-37 (PMC1779270; doi:10.1186/1477-7517-3-37)
Supplement: Additional File 1 — Peer-Reviewed Scientific/Medical Articles Acknowledging that Smokeless Tobacco Use Confers Less Risk Than Cigarette Smoking, In Reverse Chronological Order 2006-1980. [file 1477-7517-3-37-S1.doc]

**Peer-Reviewed Scientific/Medical Articles Acknowledging that Smokeless Tobacco Use Confers Less Risk Than Cigarette Smoking, In Reverse Chronological Order 2006-1980.**

1. Foulds J, Ramstrom L. Causal effects of smokeless tobacco on mortality in CPS-I and

CPS-II? [Letter to the editor]. *Cancer Causes Control* 2006; **17:** 227-8.

“[W]e agree that the health risks from use of smokeless tobacco products are

markedly smaller than those from smoking tobacco products. We believe it

would be helpful for health professionals and the public to be provided with best

estimates of the relative risks of death from all causes and specific diseases,

comparing exclusive smokeless tobacco users with exclusive smokers.”

2. Haukkala A, Vartiainen E, de Vries H. Progression of oral snuff use among Finnish 13–16-year-old students and its relation to smoking behaviour. *Addiction* 2006; **101:** 581-9.

“Although snuff use has adverse health consequences, these are clearly smaller

than with cigarette smoking.”

3. McNeill A, Bedi R, Islam S, Alkhatib MN, West R. Levels of toxins in oral tobacco

products in the UK. *Tob Control* 2006; **15:** 64-7.

“[S]mokeless tobacco users should also be informed about the much greater

health risks of cigarette smoking to prevent them switching to this more

dangerous form of nicotine delivery.”

4. Rutqvist LE , Lewin F. Flawed methods. [Letter to the editor]. *Int J Cancer* 2006; **118:**

1581.

“The consistent lack of an association between non-smoking tobacco and several

major, smoking-related cancers clearly illustrates the ‘harm reduction’ potential of

the type of smokeless products currently used in northern Europe.”

5. Thompson F, Fagerstrom K. Current trends in international tobacco control. *Clin Occup Environ Med* 2006; **5:** 101-16.

“However one interprets the Swedish experience, it is clear that Swedish snus is

substantially less hazardous than cigarettes, because users are not exposed to the

many dangerous compounds that are created by combustion.”

6. Accortt NA , Waterbor JW, Beall C, Howard G. Cancer incidence among a cohort of

smokeless tobacco users (United States). *Cancer Causes Control* 2005; **16:** 1107-15.

“In contrast to the well-known deleterious effects of cigarette smoking, ST use

did not substantially increase the risk for cancer incidence above that of nontobacco

users, particularly among males. Although the use of tobacco in any

form is to be discouraged, our data suggests that cancer risks are much lower from

ST use than from cigarette smoking.”

7. Colby SM, Drobes DJ, West R. International advances in nicotine and tobacco research. 11th Annual Meeting Society for Research on Nicotine and Tobacco, Prague, Czech Republic, 20 – 23 March 2005. *Nicotine Tob Res* 2005; **7**: 667-709.

“While complete abstinence from both tobacco and nicotine is the ideal, Dr.

Fagerström urged SRNT researchers not to let that ideal stand in the way of the

possible. He asserted that NR [nicotine replacement] and also low-nitrosamine

smokeless tobacco is by several orders so much less harmful compared with

smoking that such forms could be used to make cigarette smoking less prevalent

in our societies.”

8. Furberg H, Bulik CM, Lerman C, Lichtenstein P, Pedersen NL, Sullivan PF. Is Swedish snus associated with smoking initiation or smoking cessation? *Tob Control* 2005; **14:** 422-4.

“Swedish snus is a moist smokeless tobacco product that contains lower

concentrations of cancer-causing tobacco-specific nitrosamines than found in

other smokeless tobacco products and cigarettes. While snus delivers similar

concentrations of nicotine, it carries substantially lower risks of cancer than

cigarettes.”

9. Gray N. Mixed feelings on snus. *Lancet* 2005; **366:** 966-7.

“Is it true that snus is a harm-reduction product and an aid to quitting? Compared

with cigarettes, snus is certainly much less harmful, having, so far, not been

inculpated as actually causing any increase in lung cancer.”

10. Hergens MP, Ahlbom A, Andersson T, Pershagen G. Swedish moist snuff and

myocardial infarction among men. *Epidemiology* 2005; **16**: 12-6.

“The hypothesis that smokeless tobacco increases the risk for myocardial

infarction is not supported in the present study.”

“We found no clear evidence for an association

of snuff use with fatal infarction.”

“The difference in risks for myocardial infarction between smokers and snuff users

could suggest that it is probably not the long-term exposure to nicotine in the

smoking tobacco that increases the risk for myocardial infarction, but rather other

components in cigarette smoke. Carbon monoxide, oxidant gases, and polycyclic

aromatic hydrocarbons are substances in cigarette smoke that have a potential

cardiovascular effect. Another hypothesis would be that oral moist snuff contains

substances such as fatty acids and flavonoids that could have a protective effect

for myocardial infarction.”

11. Kozlowski LT, Edwards BQ. “Not safe” is not enough: smokers have a right to know

more than there is no safe tobacco product. *Tob Control* 2005; **14**: ii3-7.

“Finally, the ‘not safe’ or ‘not harmless’ messages don’t address the reality that

some tobacco products are substantially safer than others. Smokeless tobacco

(SLT), for example, while not safe, is substantially safer than cigarettes.”

12. Lewis S, Arnott D, Godfrey C, Britton J. Public health measures to reduce smoking

prevalence in the UK: how many lives could be saved. *Tob Control* 2005; **14**: 251-4.

“Consideration should also be given to allowing limited market freedoms to

alternative tobacco based products, such as Swedish oral smokeless tobacco

(snus), since this product has a strongly favourable profile of adverse effects

relative to cigarettes and appears to be widely acceptable as an alternative to

cigarettes, particularly to men. By 2002, 14% of Swedish male smokers had

switched from smoking to using snus, and the prevalence of smoking in Swedish

men had fallen to only 15%. This appears to have had a substantial beneficial

impact on lung cancer rates in Sweden, which are now lower than in any

comparable developed nation. Making safer nicotine sources available in the UK

as part of a controlled harm reduction strategy, even if some of these products are

not entirely risk-free, could therefore generate substantial further reductions in

smoking prevalence and related harm.”

13. O’Connor RJ, Hyland A, Giovino GA, Fong GT, Cummings KM. Smoker awareness of and beliefs about supposedly less-harmful tobacco products. *Am J Prev Med* 2005; **29**:

85-90.

“Epidemiologic data suggest that SLT products sold in the United States

are significantly less dangerous than cigarettes.”

14. Phillips C, Wang C, Guenzel B. You might as well smoke; the misleading and harmful public message about smokeless tobacco. *BMC Public Health* 2005; **5**: 31.

“The negative health consequences of smoking cigarettes are well known. What

is less well known is that not all tobacco products create similar levels of risk. In

particular, use of Western smokeless tobacco (ST) is substantially less harmful

than smoking cigarettes. This should not be surprising, given that ST use does not

expose the body to the harmful combustion products and assault on the lungs that

result from smoking. But even many health experts do not realize there is a major

difference, perhaps because of repeated messages about “tobacco” (usually

referring just to cigarettes), which imply that all products made from this plant

have the same health implications.”

“ST is usually only linked to one life-threatening disease, oral cancer (OC), and

even that association may not apply to the types of products that increasingly

dominate ST use in the West. . . . Claims are sometimes also made about links to

cardiovascular disease and pancreatic cancer, though the evidence supporting

these claims is even thinner and more equivocal. The lack of clear evidence of a

strong association with any diseases is not due to lack of research; there have been

extensive attempts to find health risks from ST, including in Swedish populations

where prevalence of use is high. While it is impossible to ever rule out small

associations between an exposure and a disease, there is ample evidence to rule

out, with a very high degree of confidence, the possibility that the combined risk

of life threatening diseases due to ST use is anything close to that from smoking.”

15. Roth HD, Roth AB, Liu X. Health risks of smoking compared to Swedish snus. *Inhal*

*Toxicol* 2005; **17**: 741-8.

“Our review of the literature indicates that, for certain health outcomes, the health

risks associated with snus are lower than those associated with smoking.

Specifically, this is true for lung cancer . . ., for oral cancer . . ., and for gastric

cancer . . . . Three of four studies showed this for cardiovascular disease . . . .

Although both snus and cigarette smoking were associated with increased risk of

all-cause mortality, the risk was significantly greater with cigarette smoking . . . .”

16. Tilashalski K, Rodu B, Cole P. Seven year follow-up of smoking cessation with

smokeless tobacco. *J Psychoactive Drugs* 2005; **37**: 105-8.

“In a previous study, the authors documented that long-term use of SLT

[smokeless tobacco] is 98% safer than smoking. According to recent research,

SLT causes neither lung cancer nor other diseases of the lung, and users have no

excess risk for cardiovascular diseases. The only consequential adverse health

effect of SLT use is oral cancer, but even this risk is minimal, and far lower than

that from smoking.”

“Data from Sweden support the role of SLT in harm reduction at the population

level. Men in Sweden have the lowest smoking rate and the highest SLT usage

rate in Europe, and Swedish men have the lowest rates of lung cancer and all

smoking-related deaths among 20 European countries.”

17. Critchley JA, Unal B. Is smokeless tobacco a risk factor for coronary heart disease? A

systematic review of epidemiological studies. *Eur J Cardiovasc Prevention Rehab* 2004;

**11**: 101-12.

“Most ST [smokeless tobacco] products are probably considerably lower risk than

cigarette smoking (taking all the potential health effects, particularly cancers, into

account). Switching to ST may reduce risks of major death and illness for some

nicotine-addicted cigarette smokers.”

18. Hatsukami DK, Henningfield JE, Kotlyar M. Harm reduction approaches to reducing

tobacco-related mortality. *Annu Rev Public Health* 2004; **25**: 377-95.

“In principle, oral noncombustible products are safer than cigarettes. They result

in exposure to fewer toxins than cigarettes, in part because there are no

combustion products, and presumably lead to less mortality and morbidity

compared to smoking. The relative reduced tobacco–related mortality and

morbidity rate in Sweden among males has been attributed to greater use of snus

as opposed to cigarettes.”

19. Hatsukami DK, Lemmonds C, Tomar SL. Smokeless tobacco use: harm reduction or

induction approach? *Prev Med* 2004; **38**: 309-17.

“Several reasons have been given for the use of ST [smokeless tobacco] as a harm

reduction method. Prominent among these reasons is the fewer negative health

consequences associated with ST compared to cigarettes. Estimates have been

made that ST poses about 2% of the mortality risk of cigarette smoking and only

half of the risk of oral cancer associated with continued cigarette smoking.”

20. Levy DT, Mumford EA, Cummings KM, Gilpin EA, Giovino G, Hyland A, Sweanor D, Warner KE. The Relative Risks of a Low-Nitrosamine Smokeless Tobacco Product Compared with Smoking Cigarettes: Estimates of a Panel of Experts. *Cancer Epidemiol Biomarkers Prev* 2004; **13**: 2035-2042.

“For total mortality, the estimated median relative risks for individual users of LN-SLT

[low-nitrosamine smokeless tobacco] were 9% and 5% of the risk associated with

smoking for those ages 35 to 49 and ≥50 years, respectively…In comparison with smoking, experts perceive at least a 90% reduction in the relative risk of LN-SLT use. The risks of using LN-SLT products therefore should not be portrayed as comparable with those of smoking cigarettes as has been the practice of some governmental and public health authorities in the past.”

“On the narrow question of the relative health risk of LN-SLT products, these results

clearly indicate that experts perceive these products to be far less dangerous than

conventional cigarettes. Based on the available published scientific literature as of

2003, there seems to be consensus that LN-SLT products pose a substantially lower risk

to the user than do conventional cigarettes. This finding raises ethical questions

concerning whether it is inappropriate and misleading for government officials or public

health experts to characterize smokeless tobacco products as comparably dangerous

with cigarette smoking.”

21. McNeill A. ABC of smoking cessation: harm reduction. *Br Med J* 2004; **328**: 885-887.

“In Sweden the use of oral moist snuff (known as snus) has been common among men

for several decades. The health risks of this product seem to be extremely low, in

absolute terms as well as in relation to cigarette smoking. Snus seems to be widely used

by smokers as an alternative to cigarettes, contributing to the low overall prevalence of

smoking and smoking related disease in Sweden.”

Snus and other smokeless oral tobacco products currently being developed by some

tobacco companies could therefore provide a viable alternative to smoking for many

smokers in other countries, and thus deliver substantial health gains. However, these

products are currently prohibited throughout the European Union (except in Sweden) on

the grounds that they are unsafe.”

22. Rodu B, Cole P. The burden of mortality from smoking: Comparing Sweden with other countries in the European Union. *Eur J Epidemiol* 2004; **19**: 129-131.

“The low smoking-related mortality among Swedish men is probably due to their use of

snus (Swedish smokeless tobacco). A recent study from northern Sweden showed that

high prevalence of snus use is strongly associated with low smoking prevalence, the

latter a result of both reduced smoking initiation and increased cessation. The

prevalence of tobacco use among Swedish men (snus use 20%, smoking 19%) is the

same as the prevalence of smoking among men throughout the EU (40%). But, because

snus use produces a very low risk for cardiovascular diseases and no risk for pulmonary

diseases and for oral or other cancers, there is no demonstrable incremental burden of

mortality among Swedish men who use snus.

Our study shows that the low prevalence of smoking among men in Sweden, if

adopted throughout the EU, would result in a 40% reduction in smoking-related

mortality.”

23. Rodu B, Jansson C. Smokeless tobacco and oral cancer: a review of the risks and determinants. *Crit Rev Oral Biol Med* 2004; **15**: 252-263.

“The available epidemiologic studies indicate that the use of chewing tobacco and

American moist snuff is associated with minimal risk for oral cancer, while the use of

Swedish moist snuff is associated with no demonstrable risk.”

24. Zatterstrom UK, Svensson M, Sand L, Nordgren H, Hirsch JM. Oral cancer after using

Swedish snus (smokeless tobacco) for 70 years – a case report. *Oral Dis* 2004; **10**: 50-53.

“From epidemiological studies it is clear that the risk of cancer to snuff dippers is nothing like as great as that in cigarette smokers.”

25. Asplund K. Smokeless tobacco and cardiovascular disease. *Prog Cardiovasc Dis* 2003 **45**: 383-394.

“[T]he use of smokeless tobacco (with snuff being the most studied variant) involves a

much lower risk for adverse cardiovascular effects than smoking does.”

26. Bates C, Fagerström K, Jarvis M, Kunze M, McNeill A, Ramström L. European Union policy on smokeless tobacco. A statement in favour of evidence-based regulation for public health. *Tob Control* 2003; **12**: 360-367.

“We believe that the partial ban applied to *some* forms of smokeless tobacco in the EU

should be replaced by regulation of the toxicity of *all* smokeless tobacco. We hold this

view for public health reasons: smokeless tobacco is substantially less harmful than

smoking and evidence from Sweden suggests it is used as a substitute for smoking and

for smoking cessation…We think it is wrong to deny other Europeans this

option for risk reduction and that the current ban violates rights of smokers to control

their own risks. For smokers that are addicted to nicotine and cannot or will not stop, it

is important that they can take advantage of much less hazardous forms of nicotine and

tobacco—the alternative being to ‘‘quit or die’’… and many die.”

“[F]or oral tobacco to play a role in harm reduction it is not necessary to show that it

does not cause cancer – it just needs to be substantially less hazardous than smoking.

Even allowing for cautious assumptions about the health impact, snus – and other oral

tobaccos – *are a very substantially less dangerous way to use tobacco than cigarettes.*

Smokeless tobaccos are not associated with major lung diseases, including chronic

obstructive pulmonary disease (COPD) and lung cancer, which account for more than

half of smoking related deaths in Europe. If there is a CVD risk, which is not yet clear,

it appears to be a substantially lower CVD risk than for smoking. Smokeless tobacco

also produces no environmental tobacco smoke (ETS) and therefore eliminates an

important source of disease in non-smokers and children. These are very substantial

benefits in reduced risk to anyone that switches from smoking to smokeless tobacco and

we believe the public health community has a moral obligation to explore this strategy.

It is likewise ethically wrong to actively *deny* users the option to reduce their risk in this

way.”

“The risk to the user arising from use of a smokeless tobacco product varies by product

and is to some extent uncertain – notably in the area of heart disease (though *at worst*

the heart disease impact appears to be substantially less than smoking). However, we

are confident that the evidence base described above and elsewhere suggests that it is

reasonable to formulate the overall relative risk as follows: *on average Scandinavian or*

*some American smokeless tobaccos are at least 90% less hazardous than cigarette*

*smoking.* In a spectrum of risk, snus is *much* closer to NRT [nicotine replacement

therapy] than it is to cigarette smoking.”

27. Britton J. Smokeless tobacco: friend or foe? *Addiction* 2003; **98**: 1199-1201.

“An alternative approach, which has already proved acceptable to smokers and to be

commercially viable, is smokeless tobacco. . . .”

“Studies of snus in particular have demonstrated relatively modest effects on oral cancer

and cardiovascular disease, few of which are statistically significant. Whilst a lack of

statistical significance clearly does not rule out important effects, it is also evident that

the risks of snus are substantially less than those of smoked tobacco.”

“The Swedish experience suggests strongly that this would reduce the current burden of disease caused by smoking.”

28. England LJ, Levine RJ, Mills JL, Klebanoff MA, Yu KF, Cnattingius S. Adverse pregnancy outcomes in snuff users. *Am J Obstet Gynecol* 2003; **189**: 939-943.

“Because the use of smokeless tobacco does not result in exposure to products of

combustion, snuff is considered to be generally less harmful than cigarettes. Compared

with cigarette smoking, snuff use appears to have less effect on atherosclerosis, risk of

myocardial infarction, and risk of death from cardiovascular disease. The effects of

smokeless tobacco on pregnancy outcomes, however, are unclear.”

29. Fagerström KO, Schildt E-B. Should the European Union lift the ban on snus? Evidence from the Swedish experience. *Addiction* 2003; **98**: 1191-1195.

“In summary, it seems clear that while smoking tobacco is the skyscraper in terms of

health risks the use of snus, although not risk-free, is a two-storey building and on a par

with risks from many other unhealthy habits or products.”

“Taken together with the epidemiological evidence that Swedish snus is considerably

less harmful than cigarettes, and that oral cancer in Sweden is less than in countries with

higher smoking rates but lower SFT [smoke free tobacco] prevalence, suggests that not

banning snus has probably been beneficial to Swedish public health.”

30. Foulds J, Ramström L, Burke M, Fagerström K. Effect of smokeless tobacco (snus) on smoking and public health in Sweden. *Tob Control* 2003; **12**: 349-359.

“As of 2000, Sweden has a lower standardised rate of male lung cancer incidence than

any comparable developed nation in the world. Sweden also has a low rate of oral

cancer by international standards and this low rate has been falling over the past 20

years while snus use has increased. . . . Interestingly Swedish men have also had a

significant improvement in cardiovascular health over the same period. . . . It is

noteworthy that these improvements in tobacco caused illnesses have occurred

primarily in men, despite a stable consumption of tobacco among men during that time

period. The main factor that has changed is that many Swedish men have switched

from smoked tobacco to snus.”

“This review suggests that snus is notably less harmful to health than cigarettes, and

that in Sweden snus has served as a pathway from smoking, rather than a gateway to

smoking among Swedish men. A proportion of the reduction in illness caused by

tobacco among Swedish men may be attributable to their shift from smoking to snus

use.”

“Snus is clearly less harmful to the individual user than smoked tobacco, and also less

harmful than the types of smokeless tobacco used in some other parts of the world,

notably Sudan and India.”

31. Gilljam H, Galanti RM. Role of snus (oral moist snuff) in smoking cessation and smoking reduction in Sweden. *Addiction* 2003; **98**: 1183-1189.

“Because most tobacco-related harm is caused by inhaled components of tobacco

smoke, unburned tobacco products are considered relatively safer. An intuitive

approach to harm reduction would therefore be to substitute inhaled nicotine with

nicotine from other sources. . . .”

“A real-life long-term test of the concept of harm reduction has been ongoing in Sweden

with little or no scientific monitoring. . . . Due to the lack of consistent associations with

major diseases, snus is considered to be less harmful than cigarettes and few users seek

help for their addiction.”

32. Kozlowski LT, O’Connor RJ. Apply federal research rules on deception to misleading health information: an example on smokeless tobacco and cigarettes. *Public Health Rep* 2003; **118**: 187-92.

“Two respected agencies of the U.S. Department of Health and Human Services

(DHHS) – the Centers for Disease Control and Prevention (CDC) and the Substance

Abuse and Mental Health Services Administration (SAMHSA) – have maintained

websites which have erroneously reported that smokeless tobacco is not safer than

cigarettes. This claim is not supported by science and acts unethically to mislead

readers of these websites.”

“Wherein lies the error or possible deception in asserting that smokeless tobacco and

cigarettes are equally dangerous? First, there is no scientific doubt that smokeless

tobacco is substantially safer than cigarettes. (The Institute of Medicine [IOM] report

provides a thorough review, indicating that smokeless tobacco is safer than cigarettes,

particulary as used in Sweden and North America, rather than as used in India.)

Smokeless tobacco does not cause respiratory disease or lung cancer; meaning that there

would be at least 60% fewer deaths from smokeless than from cigarettes. In fact,

epidemiological analyses estimate that smokeless tobacco has only 2% of the risk of

cigarettes. . . . The predominant health risk linked to smokeless tobacco is oral cancer,

but cigarettes seem to be even more strongly linked to oral cancer than is smokeless

tobacco.”

33. Kozlowski LT, O'Connor RJ, Quinio Edwards B. Some practical points on harm reduction:what to tell your lawmaker and what to tell your brother about Swedish snus. *Tob Control* 2003; **12**: 372-373.

“To provide the most useful information to consumers, information comparing the most

dangerous products (cigarettes) to much less dangerous products (snus, medicinal

nicotine) is an essential component of legislation. By limiting comparative claims only

to like products (cigarettes to cigarettes, smokeless to smokeless), the consumer is

prevented from understanding where maximal harm reduction lies.

Individuals who do use or who are thinking of using cigarettes have a right to know that

smokeless products are safer than cigarettes. . . . Snus and medicinal nicotine are so

much safer than cigarettes that net societal harm is very unlikely.”

34. McKenna JW, Pechacek TF, Stroup DF. Health communication ethics and CDC qualitycontrol guidelines for information. *Public Health Rep* 2003; **118**: 193-196.

“The CDC has issued no current public pronouncements about the relative dangers of

SLT [smokeless tobacco] and cigarettes, given the lack of scientific consensus about

this and other harm-reduction issues in tobacco control. In general, most public health

authorities agree that SLT is less hazardous than cigarette use in terms of overall

mortality when evaluated in the context of lifetime exclusive use.”

35. Ramström LM. Implications of nicotine dependence: need for revision of tobacco product regulations. *Wien Klin Wochenschr* 2003; **11**: 401– 402.

“Like all smokeless tobacco products snus is free from the combustion products that are

the main health damaging constituents of cigarette smoke. This makes it reasonable to

expect a generally lower health risk associated with this product than with cigarettes.”

36. Ramström L. Snus: part of the problem or part of the solution? *Addiction* 2003; **98**: 1198-1199.

“Since the major harmful exposure from cigarettes comes from combustion products, a great deal of interest has been given to non-combustible tobacco products, especially snus, a Sweden-specific kind of moist oral snuff. This is because Swedish men have been combining record high consumption of snus and record low levels of tobacco-related illness.”

37. Rodu B, Stegmayr B, Nasic S, Cole P, Asplund K. Evolving patterns of tobacco use in northern Sweden. *J Intern Med* 2003; **253**: 660-665.

“In summary, this study shows the major role of snus use in lowering smoking rates

amongst men in northern Sweden during the past decade. The availability of snus as an

alternative source of nicotine that is far safer than cigarettes has permitted many men to

avoid the adverse risks of long-term smoking without abstaining from tobacco

altogether.”

38. Sweanor D. Legal strategies to reduce tobacco-caused disease. *Respirology* 2003; **8**: 413-418.

“In addition, smokers who underestimate the health benefits of greatly less hazardous alternatives, such as medicinal nicotine or at least some forms of smokeless tobacco, are inadequately informed. For example, recent US research showed that 50% of smokers erroneously believed that nicotine causes cancer and 80% believed that smokeless tobacco was just as likely as cigarettes to cause cancer.”

39. Accortt NA, Waterbor JW, Beall C, Howard G. Chronic disease mortality in a cohort of smokeless tobacco users. *Am J Epidemiol* 2002; **156**: 730-737.

“Evidence from this study shows that smokeless tobacco use is a

safer alternative than continued cigarette smoking. Mortality for exclusive smokeless

tobacco users is considerably less than mortality for exclusive smokers for all cancer

(especially lung cancer) and for ischemic heart disease.”

40. Ault RW, Ekelund RB. The personal economics of smoking cessation. *J Family Consumer Sci* 2002; 94: 41-49.

“The failure to present smokeless tobacco and long-term use of nicotine replacement therapies as alternatives to smoking comes from a fear of recommending any therapy that has any harmful health consequences. However, there is no logic for arguing against a therapy that results in a net reduction in harm and economic costs.” (p. 48).”

41. Cummings KM. Can capitalism advance the goals of tobacco control? *Addiction* 2002; **97**: 957-958.

“Yet if all nicotine products were put on a risk continuum the actual difference between

smokeless and nicotine medications would be seen as fairly minor compared to the

difference in disease risk between smoked and smokeless products (Stratton et al.

2001). Until smokers are given enough information to allow them to choose products

because of lower health risks, then the status quo will remain. Capitalism, and not

government regulation, has the greatest potential to alter the world-wide epidemic of

tobacco-related disease.”

42. Kozlowski LT. Harm reduction, public health, and human rights: smokers have a right to be informed of significant harm reduction options. *Nicotine Tob Res* 2002; **4 Suppl 2**: S55-60.

“Snus reduces tobacco harm dramatically in comparison to cigarettes . . . . Since about

half of cigarette deaths arise from lung cancer and respiratory disease . . . and since

smokeless products are not otherwise more dangerous than cigarettes, smokeless

tobacco products can be estimated to reduce mortality by at least half, because they do

not cause lung cancer or respiratory disease. Snus is lower than other moist snuffs in

known toxins (N-nitrosamines and polynuclear aromatic hydrocarbons) . . . There has

been concern about smokeless tobacco and oral cancer. Noting the high rate of snus use

in Sweden and citing five studies, the Institute of Medicine report stated, ‘[T]he use of

snus in Sweden has generally not been associated with oral cavity cancer. . . .

There also are no secondhand smoke or fire risks from snus. The findings are mixed on

whether snus contributes to cardiovascular disease . . . Snus is not safe, but, on the basis

of toxicological principles (no smoke toxins from smoke exposure to the lungs) and

current epidemiological knowledge, snus is significantly less dangerous to individual

users than cigarettes.”

43. Tobacco Advisory Group of the Royal College of Physicians. Protecting smokers saving lives. A case for tobacco and nicotine regulatory authority. *Royal College of Physicians of London*. December 2002.

“As a way of using nicotine, the consumption of non-combustible tobacco is of the

order of 10-1,000 times less hazardous than smoking, depending on the product.”

44. Asplund K. Snuff – how dangerous is it? The controversy continues. (Editorial). *J Intern Med* 2001; **250**: 457-461.

“Obviously, from a medical point of view, the only reasonable norm is nonuse of

tobacco. The goal should be a tobacco-free society. On the route towards this goal, we

face enemies that are more perilous than others. My personal opinion is that we should

not squander our forces. We should concentrate them on the fight against cigarettes –

they are the overwhelming threat to people’s health, snuff being only a mini-monster in

comparison.”

45. Bates C. Clearing the smoke or muddying the water? (Editorial). *Tob Control* 2001; **10**: 87-88.

“While I do not wish to see a rush of novel tobacco products making health claims,

there are immediate real problems arising from the ‘self serving caution’ of regulators.

For example, this applies to the refusal of some regulators to approve harm reduction

indications for pharmaceutical NRT [nicotine replacement therapy], the regulatory

barriers facing a nicotine gum manufacturer that wants to compete directly with

cigarettes as a supplier of lifestyle branded nicotine, and the clumsy regulation of

certain oral tobaccos that have substantially lower health risks than cigarettes.”

46. Galanti MR, Wickholm S, Gilljam H. Between harm and dangers. Oral snuff use, cigarette smoking and problem behaviours in a survey of Swedish male adolescents. *Eur J Public* *Health* 2001; **11**: 340-345.

“The prevalence of smokeless tobacco use (moist snuff) in Sweden is among the highest

world-wide and snuff is gaining popularity as a less harmful alternative to cigarettes.”

“The use of Swedish oral tobacco (moist snuff) has not been consistently associated

with relevant health hazards, and is therefore regarded as relatively safe compared to

cigarette smoking.”

47. Galanti MR, Nordgren P, Gilljam H. “Nicotine niche” or way out? The use of oral snuff among Swedish current and former smokers. *3rd SRNT Europe Conference* 19 – 22 September

2001, p. 52.

“To date, there is no convincing evidence of major adverse health effects associated

with the use of the Swedish smokeless tobacco ‘*snus*’. It has also been claimed that

*snus* availability may have substantially contributed to lower the adult smoking

prevalence.”

48. Johnson N. Tobacco use and oral cancer: A global perspective. *J Dent Educ* 2001; **65**: 328–339.

“All forms of tobacco represent risk factors for oral cancer, but on present evidence,

snuff habits as they exist in Scandinavia and probably in the United States carry lower

risks of serious health hazards, including oral cancer.”

49. Stratton K, Shetty P, Wallace R, Bondurant S (eds.). Clearing the smoke*.* Assessing the science base for tobacco harm reduction. Institute of Medicine. National Academy Press, Washington, D.C., 2001

“Smokeless tobacco products are associated with oral cavity cancers, and a dose-response relationship exists. However, the overall risk is lower than for cigarette

smoking, and some products such as Swedish snus may have no increased risk.”

50. Warner KE. Reducing harm to smokers: methods, their effectiveness, and the role of policy. In: Rabin RL, Sugarman SD (eds.). Regulating tobacco. Oxford University Press 2001: 111-142.

“And smokeless tobacco use, although definitely not without disease risks of its own, is

unarguably less risky than smoking.”

51. Ramström L. Snuff – an alternative nicotine delivery system. In: Ferrence R, Slade J, Room R, Pope M (eds.). Nicotine and public health. The American Public Health Foundation, Washington D.C., 2000; Chapter 9: 159-178.

“While snuff use may entail some health risks, there is good evidence that these are

substantially lower than those associated with smoking. Switching from smoking to

snuff use would therefore represent a reduction in health risks.”

52. Benowitz NL. Editorial comment: Snuff, nicotine and cardiovascular disease: Implications for tobacco control. *J Am Coll Cardiol* 1999; **34**: 1791-1793.

“Rapid delivery of nicotine through cigarette smoke is expected to produce more

deleterious effects on the arterial circulation than nicotine absorbed more slowly

through the buccal mucosa, as is the case with the use of snuff.”

“Overall, the epidemiologic and experimental data suggest that nicotine absorbed from

smokeless tobacco, nicotine gum or transdermal nicotine is not a significant risk factor

for accelerating coronary artery disease or causing acute cardiovascular events.”

53. Borland R, Scollo M. Opportunities for harm minimization in tobacco control. *Drug Alcohol Rev* 1999; **18**: 345-353.

“It is known that long-term use of oral snuff, a form of tobacco used widely in Sweden

and the United States, is less harmful than tobacco smoking.”

54. Huhtasaari F, Lundberg V, Eliasson M, Janlert U, Asplund K. Smokeless tobacco as a possible risk factor for myocardial infarction: A population-based study in middle-aged men. *J Am Coll Cardiol* 1999; **34**: 1784-1790.

“The present observations would show that, from a cardiovascular perspective, the

deleterious effects of snuff dipping are much less than those of cigarette smoking.”

55. Rodu B, Cole P. Nicotine maintenance for inveterate smokers. *Technology* 1999; **6**: 17-21.

“[M]odern smokeless tobacco products have three major benefits to offer the inveterate

smoker. First, although smokeless tobacco is perceived as hazardous, it poses only

about two percent of the mortality risk of smoking. Even the risk of oral cancer—the

one consequential health effect of smokeless tobacco—is only one-half that of

continued smoking. Smokeless tobacco causes none of the other health effects of

smoking, including lung cancer, emphysema, and cardiovascular diseases.”

56. Dretchen K, Slade J, Kessler D, Koop CE, et al. Conference on tobacco dependence:

innovative regulatory approaches to reduce death and disease: selected excerpts from

conference proceedings. In: Warner KE, Peck CC, Woosley RL, Henningfield JE, Slade J, Page J. Tobacco dependence: Innovative regulatory approaches to reduce death and disease. *Food and Drug Law J Supplement* 1998; **53**: 115-137.

“I [Ken Warner] did want to mention just two interesting substances that we ought to be

thinking about as we go through this discussion. One is Snus [Swedish snuff], which

has been mentioned here previously. This is the smokeless tobacco that is in

widespread use, for many decades now, in Sweden that is very low in cancer-causing

nitrosamines, and quite high in nicotine. The evidence is that it increases health risks

compared to that of nontobacco users very little. There appears to be a slight oral

cancer risk. There is no evidence of an elevated cardiovascular risk. So this is a

population of long-term heavily dependent nicotine users who are not observing

significant health consequences over a period of decades.”

57. Fagerström KO, Ramström L. Can smokeless tobacco rid us of tobacco smoke? *Am J Med* 1998; **104**: 501-503.

“The Swedish National Board of Health and Welfare concluded in its report that ‘the

health risks related to smokeless tobacco are with great probability lower than those

related to smoking.”

58. Jimenez-Ruiz C, Kunze M, Fagerström KO. Nicotine replacement: A new approach to reducing tobacco-related harm. *Eur Respir J* 1998; **11**: 473-479.

“The practice of using snuff has not been found to significantly increase mortality. Thus, reduced smoking and/or abstinence combined with alternative nicotine delivery devices appears to be an untapped resource in the quest to reduce tobacco-related harm.”

59. Lewin F. Smokeless tobacco. In: Ramström L, Uranga R, Hendrie A (eds.). *Social and economic aspects of reduction of tobacco smoking by use of alternative nicotine delivery systems* (*ANDS*), Adis International Limited, United Nations. 1998: 10.

“In summary, the lack of health risks observed with Swedish snuff suggest that it could offer an alternative to cigarette smoking.”

60. Ramström L, Uranga R, Hendrie A (eds.). *Social and economic aspects of reduction of tobaccosmoking by use of alternative nicotine delivery systems* (*ANDS*), Adis International Limited, United Nations. 1998: 1-25.

“[I]t is now evident that the risk of death and disease is related to not only the amount

but also the nature of tobacco exposure; for example, daily cigarette smoking is far

more dangerous than occasional use of Swedish snuff.”

61. Tilashalski K, Rodu B, Cole P. A pilot study of smokeless tobacco in smoking cessation. *Am J Med* 1998; 104: 456-458.

“Smokeless tobacco is a potential alternative for inveterate smokers because

we have estimated its adverse health effects may be as low as 2% of those of smoking.”

62. Ahlbom A, Olsson UA, Pershagen G. Health hazards of moist snuff. *Socialstyrelsen Report* 1997; **11**: 3-29.

“Taken together, the data suggest that long-term use of snuff does not have any marked

effects on the major risk factors for cardiovascular disease.”

“The health hazards of snuff are in all likelihood smaller than those associated with

smoking.”

63. Bolinder G. Smokeless tobacco – a less harmful alternative? In: Bolliger CT, Fagerström KO. (eds.) *The Tobacco Epidemic: Progress in Respiratory Research*. Volume 28; Karger Publishing, Basel. 1997: 199-212.

“[C]ompared with the extremely high risk of developing cancer due to tobacco smoking

(according to the WHO, 33% of all cancers in the industrialized world are caused by

smoking), it seems as if the risks associated with the use of smokeless tobacco are

obviously of minor importance.”

“The risk of adverse health effects is evidently less serious in smokeless tobacco users

than in smokers . . . .”

64. Barker BF, Barker GJ. Oral tobacco use. (Letter to the Editor). *Oral Surg Oral Med Oral Pathol Radiol Endod* 1996; 81: 132.

“It cannot be denied that serious diseases and death rates associated with the use of

tobacco are far greater for those who smoke cigarettes than for those who use smokeless

tobacco.”

65. Dunlap C. Smokeless tobacco. (Letter to the Editor). *Oral Surg Oral Med Oral Pathol Radiol Endod* 1996; 81: 376.

“The central theme [of Vigneswaran’s article, Tobacco use and cancer,] was that

smokeless tobacco use is far safer than other forms of tobacco use. To me, the theme

rings true… Although I don’t approve of smokeless tobacco use, I think the carcinogenicity of smokeless tobacco has been overstated and the authors are essentially correct in their assessment of this issue.”

66. Rodu B, Cole P. The rewards of smoking cessation. *Epidemiology* 1996; 7: 111-112.

“Lifelong smokeless tobacco use has little impact on life expectancy, and this analysis

shows that switching to smokeless tobacco has essentially the same effect on life

expectancy as does quitting smoking.”

67. Vigneswaran N, Tilashalski K, Rodu B, Cole P. Oral tobacco use. (Letter to the Editor). *Oral Surg Oral Med Oral Pathol Radiol Endod* 1996; 81: 133.

“In fact, the health risks related to ST use are only 2%

those of smoking, and the smoker who switches reduces risks for *all* smoking-related

illnesses. . . .”

“Actual data show that the products of tobacco combustion, but not necessarily nicotine,

are associated with deaths from hypertension, ischemic heart disease, cerebrovascular

diseases, and atherosclerosis, whereas ST use is not associated with mortality from

myocardial infarction. . . .”

“[T]he scientific and medical foundation for smokers switching to ST is solid. The

average lifelong smoker loses almost 8 years of life compared with the nonuser of

tobacco, whereas the ST user’s loss is 15 days.”

68. Vigneswaran N, Tilashalski K, Rodu B, Cole P. Smokeless tobacco. (Letter to the Editor). *Oral Surg Oral Med Oral Pathol Radiol Endod* 1996; 81: 377.

“Our proposal [that inveterate cigarette smokers switch to smokeless tobacco], however,

has a clear and legitimate scientific basis:

1. The risk of oral cancer from smokeless tobacco use is only half that from

smoking.

2. Smokeless tobacco use carries no excess risk for other cancers or for lung

and heart diseases that kill over 400,000 smokers every year.

3. Smoking is the most dangerous nicotine delivery system. Lifelong cigarette

smokers lose almost 8 years of life on average compared with nonusers

of tobacco: in contrast, smokeless tobacco users lose only 15 days.”

69. Tilashalski K, Lozano K, Rodu B. Modified tobacco use as a risk-reduction strategy. *J Psychoactive Drugs* 1995; 27: 173-175.

“A novel approach for smoking cessation has been proposed: that cigarette smokers

unable or unwilling to quit change to smokeless tobacco (ST) use. In fact, surveys

reveal that as many as 7% of former smokers have already modified their tobacco use to

ST, which they perceive as less risky despite repeated warnings by medical authorities.

Data indicate that former smokers who now use ST have indeed chosen a safer mode of

tobacco use that serves as a positive risk-reduction strategy.”

70. Vigneswaran N, Tilashalski K, Rodu B, Cole P. Tobacco use and cancer. *Oral Surg Oral Med Oral Pathol Radiol Endod* 1995; 80: 178-182.

“Previous research has demonstrated that the relative risk of oral cancer with smokeless

tobacco use is 4.2, about half of the risk from smoking (relative risk = 10 to 15).

Mortality data from populations with sustained high-frequency smokeless tobacco use

do not support the mistaken prediction of an epidemic of oral cancer with increasing

smokeless tobacco use. In fact, the risks of smokeless tobacco use compare so

favorably with those of smoking that smokers who switch to smokeless tobacco reduce

their risks for all tobacco-related illnesses including oral cancer. Although some

criticize this proposal as less than an ideal solution for the nation’s smokers, full

adoption of this strategy would eventually save over 400,000 lives each year.”

71. Rodu B. An alternative approach to smoking control. *Am J Med Sci* 1994; 308: 32-

34.

“The author . . . proposes that smokeless tobacco be recommended as a cigarette

substitute by persons who cannot stop smoking. This proposal is made because

smokeless tobacco is associated with far fewer and considerably less serious health

consequences than is smoking. . . .”

“Because smokeless tobacco causes far fewer and considerably less serious health

effects than does smoking, it should be promulgated as an alternative to cigarettes for

smokers unable or unwilling to overcome their nicotine addiction.”

72. Rodu B, Cole P. Tobacco-related mortality. *Nature* 1994; 370: 184.

“We suggest that abstinence is not the only approach to reducing tobacco-related

mortality: for smokers addicted to nicotine who would not otherwise stop, a permanent

switch to smokeless tobacco could be an acceptable alternative to quitting.”

73. Kozlowski LT. Reduction of tobacco health hazards in continuing users: Individual behavioral and public health approaches. *J Substance Abuse* 1989; 1: 345-357.

“There is no doubt that smokeless tobacco products are gram for gram less hazardous

than smoked tobacco products. Smokeless tobacco products present no fire risks. . . .

No added doses of smoke toxins (e.g. carbon monoxide, tar) get into the lungs of the

user of oral or nasal snuff. There are no apparent lung cancer risks of using smokeless

tobacco. It may be appropriate here to emphasize again that I do not suggest that

smokeless tobacco products are safe. Nonetheless, these products are unquestionably

less risky than smoked tobacco products.”

74. Peto R. Control of tobacco-related disease. In: *The Value of Preventive Medicine.* The Ciba Foundation Symposium 110. Pitman, London. 1985; pp. 126-142.

“If this [snuff dipping] or some other such habit were to become widespread and did to

any substantial extent replace smoking (particularly of cigarettes), then the net effect

would be likely to be a reduction in tobacco-induced mortality. For, although snuff

dipping causes a vast increase in the *relative* risk of cancer of the gum and cheek

(together with the same sort of risks of cancers of other parts of the mouth that smoking

produces), the *absolute* excess risks of death from oral cancer associated with the habit

in the South-Eastern United States appear to be at most a few per cent of the total risk of

death produced by cigarette smoking. Although the absolute risks in other populations

might, of course, be considerably different (especially if some diseases other than oral

cancer are found to be increased by tobacco ‘dipping’) the use of smokeless tobacco is

still likely to be much less hazardous than is tobacco smoking, especially of cigarettes.”

75. Russell MAH, Jarvis MJ, West RJ, Feyerabend C. Buccal absorption of nicotine from

smokeless tobacco sachets. (Letter to the Editor). *The Lancet* 1985; **1**: 1370.

“Unlike tobacco smoking, use of wet snuff carries no risk of lung cancer, bronchitis, or

emphysema, and no risk of cardiovascular disease has been demonstrated.”

“If all smokers in Britain switched to [smokeless tobacco] sachets about 50 000

premature deaths per year might eventually be saved at an annual cost of less than 1000

deaths from mouth cancer.”

76. Kozlowski LT. Less-hazardous tobacco use as a treatment for the “smoking and health” problem. In: Smart RG, Cappell HD, Glaser FB, Israel Y, Kalant H, Popham RE, Schmidt W, Sellers EM (eds.) *Research Advances in Alcohol and Drug Problems*. Volume 8. Plenum Press, New York; 1984: Chapter 11, pp. 309-329.

“This chapter will argue that the use of less-hazardous tobacco, if prohibitionistic

impulses can be put aside, may have an important role in the treatment of the smoking

and health problem. . . . The phrase ‘less-hazardous tobacco use’ is meant to be

inclusive. Cigarettes, for example, are the most hazardous tobacco products overall. . . .

On the other hand, some less-hazardous tobacco products are less-hazardous in certain

respects no matter how they are used: chewing tobacco, for example, carries no risks of

fire and essentially no risk of lung disease.”

“There is really no dispute about whether smokeless tobaccos present fewer hazards to

the user than do smoking tobaccos. Smokeless tobaccos expose the lungs to essentially

no tobacco toxins. No carbon monoxide and no tar is produced. The oral cancers

associated with oral smokeless tobaccos are substantially less lethal and are more easily

diagnosed than lung cancers. In addition, smokeless tobaccos pose no problems of

second-hand smoke and no risks of fire.”

77. Kozlowski LT. Pharmacological approaches to smoking modification. In: Matarazzo JD, Weiss SM, Herd JA, Miller NE, Weiss SM (eds.) *Behavioral Health. A handbook of health* *enhancement and disease prevention*. John Wiley & Sons, New York; 1984: Chapter 45, pp. 713-728.

“Some less hazardous forms of tobacco use (smokeless tobaccos, pipes, cigars) are

pharmacotherapies that can be self-applied easily by those who are poorly motivated to

modify their smoking.”

“The use of smokeless tobaccos is not associated with lung cancer, chronic obstructive

lung disease, or bronchitis. Oral smokeless tobaccos are associated with oral cancers,

however, and manufactured nasal snuff may cause nasopharyngeal cancer, although

direct evidence is lacking for such an effect.”

78. Russell MAH, Jarvis MJ, Devitt G, Feyerabend C. Nicotine intake by snuff users. *Br Med J* 1981; **283**: 814-817.

“Unlike tobacco smoke, snuff is free of tar and harmful gases such as carbon monoxide

and nitrogen oxides. Since it cannot be inhaled into the lungs, there is no risk of lung

cancer, bronchitis, and emphysema.”

“Switching from cigarettes to snuff would substantially reduce the risk of lung cancer, bronchitis, emphysema, and possibly coronary heart disease as well, at the cost of a slight increase in the risk of cancer of the nasopharynx (or oral cavity in the case of wet snuff). Another advantage of snuff is that it does not contaminate the atmosphere for non-users.”

79. Kirkland LR. The nonsmoking uses of tobacco. (Letter to the Editor). *NEJM* 1980; 303: 165.

“Rather than oppose the advertising of ‘smokeless tobacco,’ I urge physicians to encourage snuff and chewing tobacco as alternatives to cigarettes. No one disputes the role of snuff and similar products as the cause of oral leukoplakia, but there is a vast difference in the frequencies of oropharyngeal carcinoma and cigarette-induced lung cancer. If five new ’dippers’ are created to avert creation of one new cigarette smoker, then progress has been made.”
